# Activation of Solid-State Emission and Photostability through Molecular Confinement: The Case of Triptycene-Fused Quinacridone Dyes

**DOI:** 10.1021/acs.orglett.3c02093

**Published:** 2023-08-28

**Authors:** Giovanni Preda, Andrea Aricò, Chiara Botta, Davide Ravelli, Daniele Merli, Sara Mattiello, Luca Beverina, Dario Pasini

**Affiliations:** † Department of Chemistry and INSTM, University of Pavia Via Taramelli 12, 27100 Pavia PV, Italy; ‡ SCITEC−CNR, Consiglio Nazionale delle Ricerche, Istituto di Scienze e Tecnologie Chimiche ‘G. Natta’, Via A. Corti 12, 20133 Milano, Italy; § Dipartimento di Scienza dei Materiali, Università degli Studi Milano-Bicocca and INSTM, Via R. Cozzi 55, 20125, Milano, Italy

## Abstract

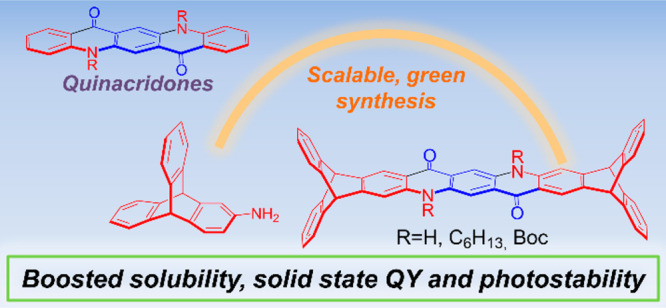

We report the facile, metal-free convergent synthesis and the characterization of novel quinacridone dyes in which two triptycene units end-cap and sterically confine the quinacridone chromophore. A precise comparison of the confined dyes with their known homologues reveals that the reduction of π–π interactions in triptycene-fused quinacridone dyes compared to classical quinacridone results not only in an increase of solubility and processability but also in an enhancement of fluorescence quantum yield and photostability in the solid state.

Organic π-conjugated systems, are currently used in a variety of optoelectronic devices because of their unique photophysical properties.^1^ The main strategy in order to tune their spectroscopic properties (absorption and luminescence) is extending or changing, by means of chemical manipulation, the π-conjugation of an aromatic system. However, in most cases, the extension of the π-system results in increased π–π stacking interactions, ultimately causing decreased solubility, with consequent problems regarding compound processability and spectroscopic performances.

An example that illustrates these difficulties well is the world-renowned, high-performance pigment quinacridone,^2^ used because of its exceptional color and weather fastness in paints, industrial inks, and artistic paintings.^3^ Quinacridone and derivatives exhibit high quantum yields in dilute solutions (∼90%–100%) and good conductivity properties, making them particularly attractive for use in optoelectronic systems.^4,5^ However, the strong intermolecular NH···O=C hydrogen bonding and pronounced π-stacking interactions of the extended π -conjugate system limit its solubility, processability, and optoelectronic properties such as emission in the solid state.^6^

The development of new strategies to overcome these inconveniences is still a real challenge. The strategies reported to date are the introduction of long alkyl and/or alkoxy chains on the quinacridone nitrogen atoms (to eliminate intermolecular hydrogen bonding),^4,7−10^ the introduction of bulky groups,^11^ (to suppress π–π-stacking interactions) or a combination of the two.^12^ In 2013, Wang and co-workers^11^ reported the synthesis of a quinacridone bearing two pentaphenyl substituents: compound **1** was found to emit efficiently from solid thin films (in contrast to pristine quinacridone). In 2015, Fang and co-workers^12^ successfully reported the synthesis the indene-fused quinacridone derivative **2** having high solubility despite preservation of intermolecular hydrogen bonds (Figure 1).

**Figure 1:**
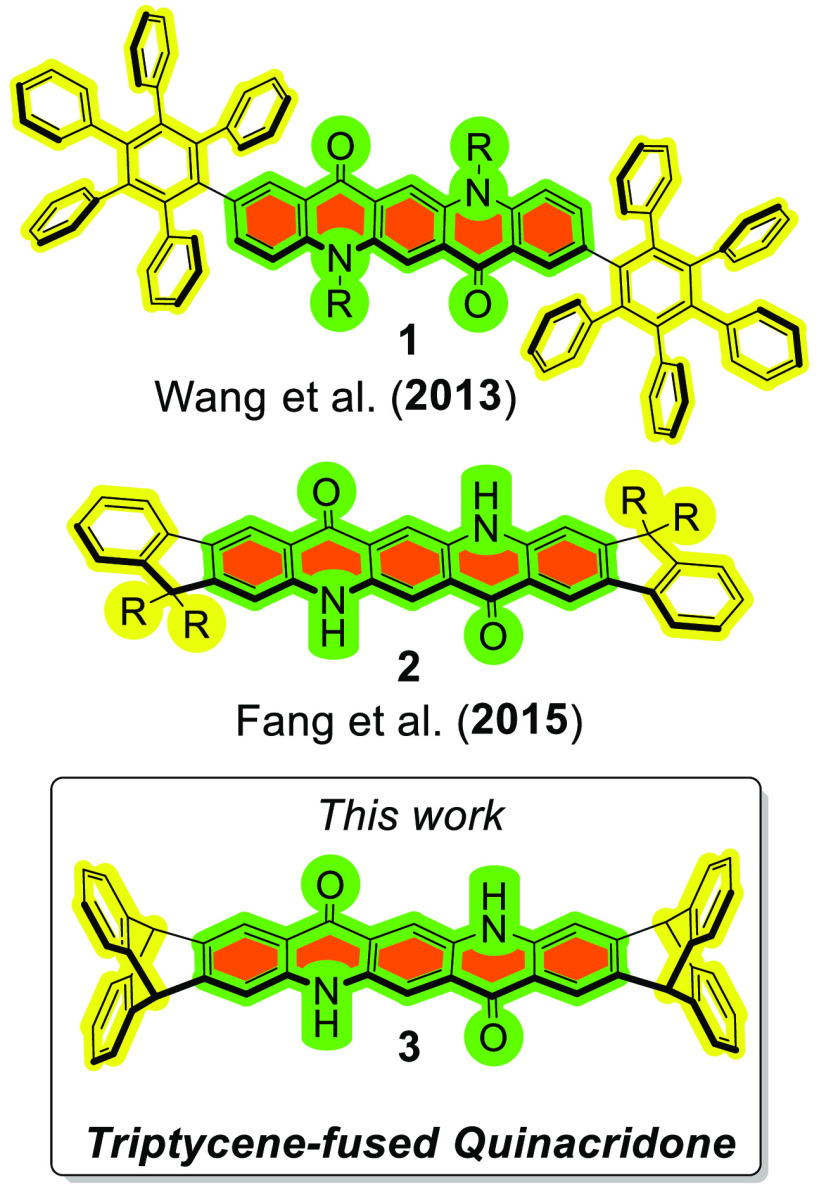
Molecular confinement strategies (top) recently published for quinacridone derivatives and (bottom) this work.

Intrigued by these studies and pushed by our recently started activity in the field,^13,14^ we decided to design systems capable of “embedding” the quinacridone chromophore within two triptycene units. Triptycene is an interesting nonplanar aromatic synthon possessing unique and fascinating characteristics and its use in the fields of supramolecular and materials chemistry is already established.^13−21^

In our expectations, the introduction of a bulky group such as triptycene should suppress π–π interactions both in solution and in the solid state, thus allowing improved solubility and luminescence efficiency while in the solid state.

Detailed studies on the solubility enhancement of π-extended chromophores by their insertion into two triptycene portions have been reported by Mastalerz and co-workers (the so-called triptycene end-capping strategy).^22−27^ However, the use of triptycene scaffold as a tool to improve the solid-state luminescence (a critical parameter for optoelectronic applications) of π-extended chromophores is still poorly investigated.

In this paper, we report the synthesis and photophysical characterization of a new class of triptycene-fused quinacridone dyes. For an accurate comparison, the new triptycene-fused quinacridone derivatives were compared with “classical” quinacridones, that were also synthesized according to procedures reported in the literature.^37,39^ We show that the embedding of quinacridone in two triptycene portions (triptycene end-capping strategy) allows the increase of *both* the solubility and processability *and* properties in the solid state, such as the fluorescence quantum yield (cast thin films) and photostability in PMMA films.

Scheme 1 shows the synthesis of all new triptycene embedded quinacridone derivatives. 2-Amino triptycene **4**, synthesized according to a procedure reported from MacLachlan,^28,29^ was condensed with dimethylsuccinylsuccinate **5**. After extensive optimization, we found that the enamine **6** can be prepared in high yield using a 1/1 ethanol/acetic acid mixture as the reaction solvent. Oxidation of **6** with molecular iodine gave **7** in an overall yield of 77%, with respect to **4**. Compound **7**, being a 2,5-diarylamino-terephthalate,^30−33^ revealed interesting photophysical properties on its own. In fact, it appeared highly fluorescent in the solid state; the fluorescence quantum yield of **7** as a powder (13%) is higher than that in solution of CHCl_3_ (5%) (see Table 1 (presented later in this work) and the electronic Supporting Information (SI)). After the quantitative basic hydrolysis of the methyl esters, the intramolecular aromatic electrophilic substitution to give the triptycene-fused quinacridone **3** dye was conducted using either poly(phosphoric acid) (PPA) or methanesulfonic acid (MSA), with the latter affording higher yields (80%), probably as a consequence of its efficient solubilizing ability of the final product.

**Scheme 1:**
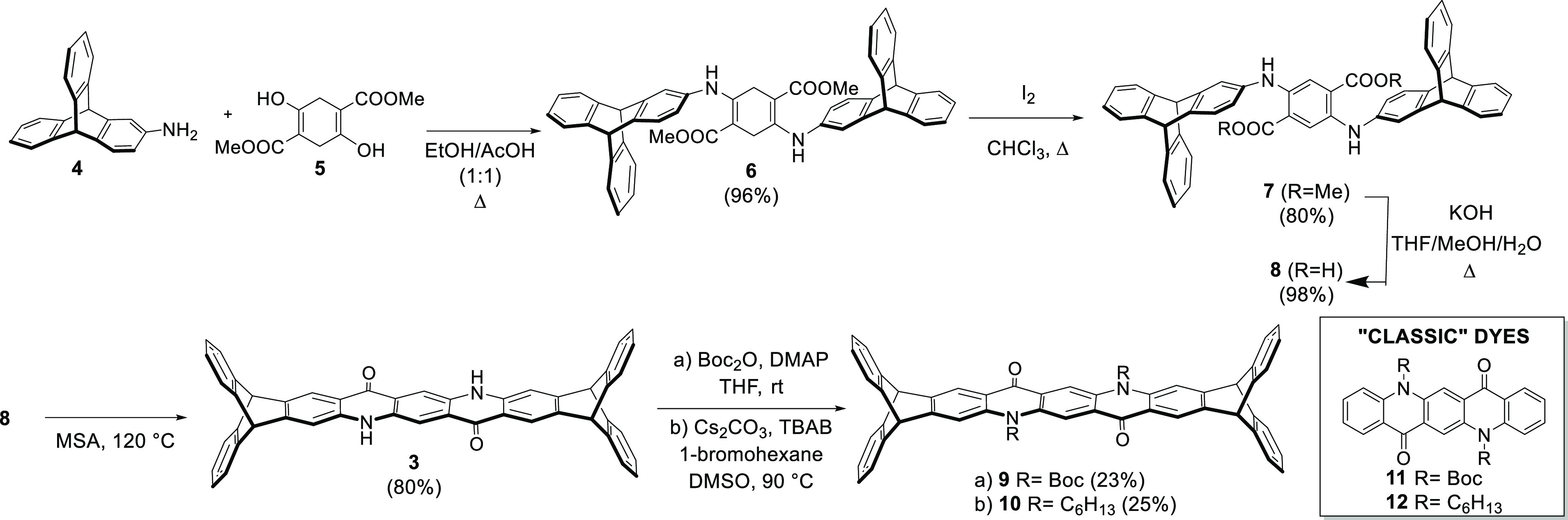
Synthesis of Triptycene-Fused Quinacridone Dyes

The comparison of the ^1^H NMR spectra of the crude and purified **3** shows that the electrophilic intramolecular cyclization of **8** is highly regioselective and nearly quantitative (Figure S1 in the Supporting Information). The regioselectivity toward position 3 of the triptycene scaffold observed in this step is consistent with reports in the literature of aromatic electrophilic substitutions carried out on the triptycene skeleton.^34^ All new compounds have been fully characterized (see the ESI). The metal-free synthetic route from 2-aminotriptycene **4** to the new triptycene-quinacridone dye **3** proposed here proved to be robust, scalable (all four synthetic steps require simple filtrations instead of chromatographic purifications) and avoided toxic and/or dangerous reagents, such as high boiling solvents (Dowtherm A or α-chloronaphthalene) or oxidizing agents (nitrobenzene or chloranil) previously reported for analogous systems.^7,35,36^ Boc and *N*-alkyl derivatives were synthesized by treatment with di-*tert*-butyl dicarbonate with 4-dimethylaminopyridine (DMAP) as the activator and 1-bromohexane in phase transfer catalysis conditions, in the presence of tetrabutylammonium bromide (TBAB), respectively (see Scheme 1).

The corresponding quinacridone derivatives **11** and **12** (Scheme 1), to be used for an accurate comparison of the spectroscopic properties of our new triptycene-quinacridone dyes, were synthesized according to reported procedures.^37,38^

A series of techniques were used for the comparison between the triptycene embedded and parent quinacridone dyes: UV–vis and photoluminescence (both in solution and as a film/powder) spectroscopies and cyclic voltammetry. The experimental results were corroborated by DFT calculations. Figure 2 shows a comparison between the optical features of the new and reference quinacridone dyes. The absorption spectra of N-alkylated quinacridone **12** (solid green line) and the corresponding triptycene derivative **10** (solid blue line) show almost superimposable π–π* HOMO–LUMO transitions, peaking at 522 and 524 nm, respectively. Conversely, the high energy portion of the spectrum shows a sizable blue shift of **12** (λ_max_ = 298 nm) with respect to **10** (λ_max_ = 318 nm). The absorption spectra of the N-Boc derivatives **9** (solid red line) and **11** (solid black line) are also very similar, both featuring a significantly blue-shifted HOMO–LUMO transition with respect to the alkylated derivatives, which is reasonable and in agreement with the electron-withdrawing nature of the Boc group on the two nitrogen atoms, reducing their ability to donate electrons into the system and therefore enhancing the HOMO–LUMO energy difference in these molecules.

**Figure 2:**
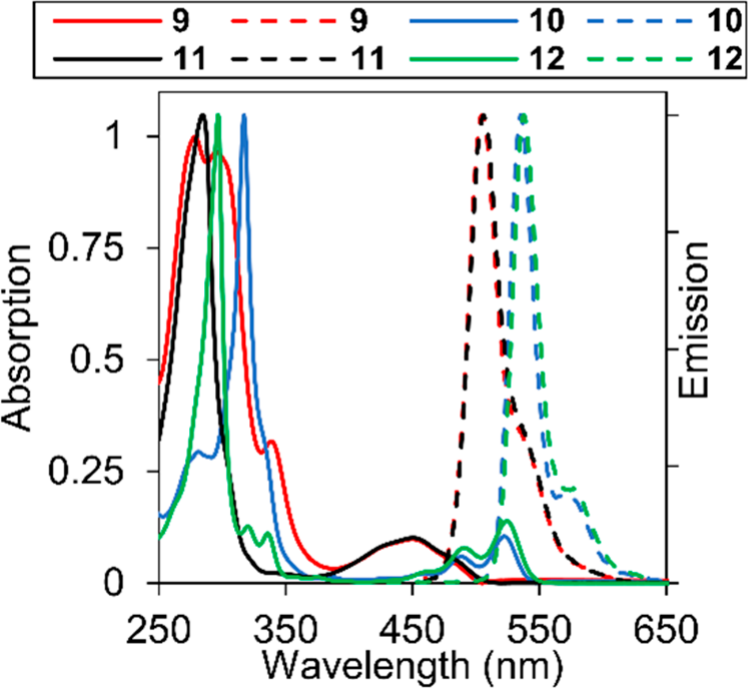
Absorption (solid lines) and emission (dotted lines) spectra in chloroform (0.5–5) × 10^–5^ M) for **9**, **10**, **11**, and **12**.

The photoluminescence properties in solution remained essentially intact: no relevant shifts between the emission maxima of **10** (532 nm) and **12** (538 nm) are present, and the fluorescence quantum yields in solution also remain essentially the same (see Table 1). Similar considerations can be made for the **9**–**11** pair (emission maxima at 503 and 505 nm, respectively). Cyclic voltammetry experiments (Table S1 in the ESI) show that the effect of the triptycene embedding in derivative **10** leads to a decrease in the oxidation potential of 0.23 eV with respect to **12**. Indeed, the energy levels of compounds **9** and **11** are almost perfectly aligned.

**Table 1:** Absorbance and Emission Propertied of Precursor **7** and Triptycene Confined Dyes **9**–**12**

compound	λ_abs_^a^^,^^c^	λ_em_^b^^,^^c^^i^	Φ_sol_^c^^,^^d^	τ^c^^,^^e^	λ_abs_^a^^,^^f^	λ_em_^b^^,^^f^^,^^i^	Φ_film_^d^^,^^f^	τ^e^^,^^f^^,^^g^
**7**	479	603 (108)	5	1.37	478	606 (66)	13^h^	3.45
**9**	449	503 (33)	94	16.8	444	533 (83)	22	5.62
**10**	522	532 (24)	84	18.6	520	548 (102)	0.5	0.58
**11**	451	505 (29)	86	16.5	457	546 (92)	1.4	1.00
**12**	522	538 (25)	90	17.5	523	590 (93)	<0.1	1.20

a Maximum absorption wavelength (nm)

b Maximum emission wavelength (nm).

c Values obtained in solution (CHCl_3_, ca. 1 × 10^–5^ M).

d Fluorescence quantum yield (%).

e In nanoseconds (ns).

f From solid film (cast film from chloroform).

g Average lifetimes (see the ESI).

h From powders.

i fwhm = full width at half-maximum.

The analyses of the frontier molecular orbitals, calculated via density functional theory [DFT B3LYP/6-311+G(2d,2p)], give further insight into the electronic structure of the new derivatives. The optimized structures of **3** and quinacridone both feature a planar π conjugated portion, with very similar electronic distribution of the corresponding frontier orbitals (Figure S33 in the SI). The geometrical structure and electronic distribution of **10** and **12** are also very similar, with only minor deviations from planarity for the latter. Conversely, the optimized structure of N-Boc triptycene derivative **9** is significantly more distorted than that of **11**, resulting in small differences in the energy and distribution of the frontier orbitals. Calculations suggest that the HOMO of **9** is partially distributed on the benzenes of the triptycene portions, while the LUMO appears similar to that of **11** (Figure S34 in the SI). All these observations suggest that the electronic structure of the quinacridone core within triptycene-fused quinacridones remains essentially unaltered, compared to classical quinacridone pigments and the contribution of homoconjugation between the triptycene and the quinacridone core is irrelevant, with respect to photophysical properties. Significant improvements could instead be observed considering solubility: **3** was soluble in THF/MeOH and DCM/MeOH mixtures, in contrast to its classical counterpart quinacridone, which was insoluble in THF or THF/MeOH mixtures.

Remarkably, quantum yield analysis associated with solid-state (film cast) emission showed that triptycene end-capped dyes are significantly more efficient than the classical counterpart. As can be seen from Table 1, **10** exhibited a significantly higher quantum yield (0.5%) than **12** (<0.1%) while **9** showed the best quantum yield (22%) vs 1.4% for **11**. Probably, the steric hindrance due to the triptycene portions of **10** is not enough to prevent solid-state packing, thus giving modest quantum yields in the solid state.

The boosted solid-state fluorescence quantum yields of triptycene-fused quinacridones compared to classical quinacridones confirmed the validity of the molecular confinement approach for the enhancing of luminescent properties, through a reduction of π–π-stacking interactions caused by the bulky triptycene units.

Photostability is another very important feature of high-performance dyes. In the past, some of us demonstrated that the use of **11** enables the preparation of doped poly(methacrylate) (PMMA) samples by the method of cell cast polymerization.^37^ The Boc-protection can be cleaved in situ after the polymerization by thermal treatment,^39^ thus leading to quinacridone doped PMMA samples, which are of interest for luminescent solar concentrators. In our previous study, the use of a commercial stabilizer was needed to sizably enhance photostability over time, as demonstrated comparing the photostability of PMMA samples doped with **Q-Alk** (analogous of **12**, having ethylhexyl chains), **QA** (quinacridone), and quinacridone stabilized with a widely used commercial compound (Figure 3). We repeated the same study by preparing cell cast PMMA samples doped with **9**. As shown in the ESI, thermal treatment of the PMMA samples at 100 °C for 66 h leads to the in situ quantitative conversion of **9** into **3**. The sample doped with **3** showed a photostability at least comparable to, if not better than, that of the stabilized quinacridone sample (Figure 3). To our knowledge, this report represents one of the few cases in which the triptycene end-capping strategy has been specifically applied to a π-extended chromophore to enhance emission and photostability performance in the solid state, and on this basis such strategy can possibly be generalized to other dyes/chromophores.^40^

**Figure 3:**
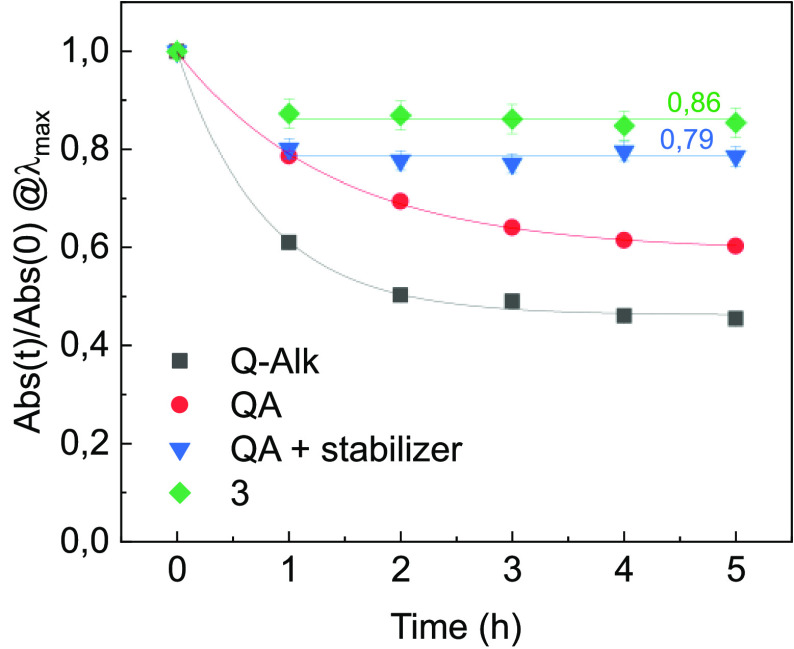
Comparison between the light-induced degradation under Solarbox irradiation at 2 Suns of PMMA samples doped with **QA** (red), **Q-Alk** (analogous of **12**, black), and **3** (green). The blue data refer to a slab containing **QA** and the commercial stabilizer bis(1octyloxy-2,2,6,6-tetramethyl-4-piperidyl) sebacate.

In conclusion, a new triptycene-fused quinacridone dye **3** was obtained by a simple synthetic scheme with good yields. From this molecule, derivatives **9** and **10** were synthesized, and for accurate and precise comparison, their photophysical properties were compared with their classical counterparts **11** and **12**. These comparisons showed, in addition to the increase in solubility, a remarkable enhancement in fluorescence quantum yield from the solid state for the triptycene-end-capped derivatives, reaching a value of 22% for film cast from solutions of derivative **9**. Furthermore, **9** can be used in the preparation of doped poly(methacrylate) (PMMA) samples by the method of cell-cast polymerization with excellent photostability. Given the increasing interest in emissive chiral molecules,^41^ and the possibility to implement chiral triptycenes,^14^ we are now exploring the application of this synthetic strategy toward chiral small-molecules^42^ and chiral helical ladder polymers^43−45^ with interesting chiroptical properties.

## Data Availability

The data underlying this study are available in the published article and its Supporting Information.

## Supporting Information Available

Experimental procedures, characterization data for all the products (PDF)
FAIR data, including the primary NMR FID files, for compounds **3** and **7**–**10** (ZIP)
