# Supplementary material for: Activation of Solid-State Emission and Photostability through Molecular Confinement: The Case of Triptycene-Fused Quinacridone Dyes
Source: Org Lett. 2023 Aug 28;25(35):6490–4. doi: 10.1021/acs.orglett.3c02093 (PMC10496147; doi:10.1021/acs.orglett.3c02093)
Supplement: Supplementary file 1 — ol3c02093_si_001.pdf [file ol3c02093_si_001.pdf]

Supporting information for:

Activation of Solid-State Emission and Photostability through Molecular Confinement: the Case of Triptycene-Fused Quinacridone Dyes

Giovanni Preda,[a] Andrea Aricò,[a] Chiara Botta,[b] Davide Ravelli,[a] Daniele Merli,[a] Sara Mattiello,[c] Luca Beverina[c] and Dario Pasini[a,*]

[a]*Department of Chemistry and INSTM, University of Pavia Via Taramelli 12, 27100 Italy;* [b]*SCITEC-CNR, Consiglio Nazionale delle Ricerche, Istituto di Scienze e Tecnologie Chimiche 'G. Natta', Via A. Corti 12, 20133 Milano, Italy;* [c]*Dipartimento di Scienza dei Materiali, Università degli Studi Milano-Bicocca and INSTM, Via R. Cozzi 55, 20125, Milano, Italy*

Table of Contents

1. *Synthesis* S2

2. *¹H-NMR comparison of commercial quinacridone and* **3** S10

3. *Uv-vis and emission studies* S11

4. *Cyclic voltammetry* S23

5. *TGA of derivative* ***9*** S25

6. *Characterization in PMMA slabs* S26

7. *DFT studies* S30

8. *Copies of NMR and mass spectra of new compounds* S43

9. *Additional references* S56

1. Synthesis

General Experimental. Commercial quinacridone was purchased from TCI (purity> 93%). All other commercially available reagents and solvents were purchased from Sigma-Aldrich, Fluorochem, TCI and Alfa Aesar. They were all used as received. Anhydrous solvents such as THF and dichloromethane (DCM) were obtained by conventional methods through distilling with certain drying agents (Na for THF and CaH_2 for dichloromethane). Flash chromatography was carried out using Merck silica gel 60 (pore size 60 Å, 270-400 Mesh). ^{1}H and ^{13}C NMR spectra were recorded from solutions in deuterated solvents on 300 Bruker or 400 Jeol spectrometers with the residual solvent as the internal standard. Mass spectra of pure compounds were recorded using an Electron Spray Ionization Agilent Technologies mass spectrometer, Direct Exposure Probe mass spectrometer, GC-MS ThermoScientific spectrometer and a Bruker Autoflex MALDI-TOF in positive mode with trans-2-(3-(4-tert-Butylphenyl)-2-methyl-2-propenylidene)malononitrile (DCTB) as the matrix. The spectra were recorded also without the matrix, giving in most cases equivalent results due to the direct formation of the radical cation of the relevant species.

Synthesis of known compounds

Triptycene

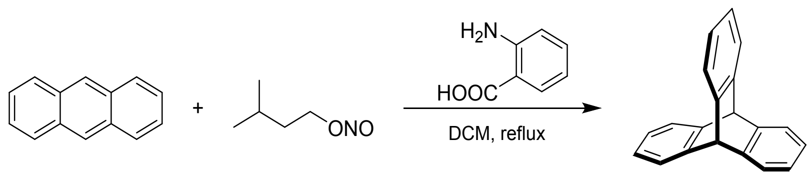

The compound was synthesized slightly modifying a procedure reported by Granda and coworkers,[S1] anthracene (20.0 g, 112. mmol) was added to a three-necked round bottom flask along with DCM (320 mL). Isopentylnitrite (19.6 mL, 146 mmol, 1.3 eq) was then added and the mixture was heated using an oil bath to reflux. A solution of anthranilic acid (16.1 g, 118 mmol, 1.05 eq) in acetone (80 mL) was added dropwise via a funnel to the reaction mixture during 3 h (during which time a gradual transition of the color to black was observed). Once the addition was complete, the reaction was allowed to reflux for 40 min, allowed to cool to room temperature and the solvent was removed *in vacuo*. Maleic anhydride (16 g, 162 mmol, 1.44 eq) in xylene (160 mL) was added to the resulting residue, and the reaction mixture was heated to reflux using an oil bath at 148 °C for 1 h. The mixture was allowed to cool at room temperature and the xylene was removed *in vacuo*. The remaining slurry was partitioned in DCM (300 mL) and 1 M NaOH (200 mL), and the organic phase treated with multiple NaOH washes. The organic phase was then collected, dried (Na_2SO_4), and filtered into a flask, from which the solvent was removed *in vacuo*. The remaining solid was suspended in EtOH,

filtered over Buchner and washed with EtOH (1 mL). A beige-colored solid (19.6 g, 38%) was obtained. Spectroscopic data in agreement with those reported in the literature. ^{1}H NMR (200 MHz, $CDCl_3$) δ = 7.42 (m, 6H), 7.03 (m, 6H), 5.47 (s, 2H).

2-Mononitrotriptycene

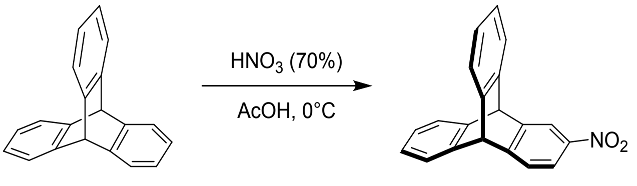

Following a procedure reported by MacLachlan and coworkers,[S2] concentrated HNO_3 65% (60 mL) was added slowly to a suspension of triptycene (5.00 g, 19.7 mmol) in glacial acetic acid (150 mL) cooled at 0°C by an ice bath. Once the addition was completed, the mixture was heated to 70 °C using an oil bath and kept refluxing for 6 h (the mixture turned light orange). Once cooled to room temperature, the mixture was quenched in a beaker containing H_2O (500 mL) and ice and left under stirring. The precipitated solid (light yellow) was filtered over Buchner and allowed to dry in an oven at 70 °C for 6 hours. The crude sample was then purified by silica gel column chromatography starting with an eluent mixture of DCM/Hex 1/1 to a 3/1 yielding 2-nitrotripticene (3.9 g, 66%). Spectroscopic data were in agreement with literature. ^{1}H NMR (200 MHz, $CDCl_3$) δ = 8.23 (d, J = 2.2 Hz, 1H), 7.94 (dd, J = 8.1, 2.2 Hz, 1H), 7.51 (d, J = 8.2 Hz, 1H), 7.53 – 7.37 (m, 4H), 7.06 (m, 4H), 5.55 (s, 2H).

2-aminotriptycene

Route A

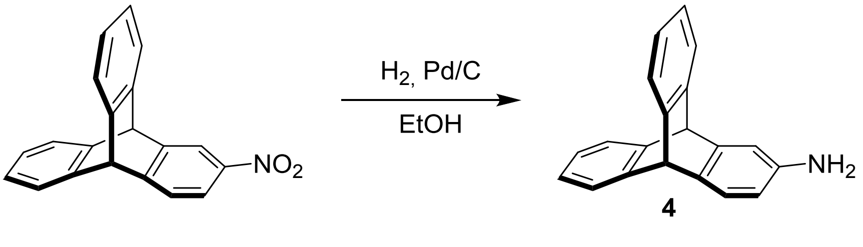

Following a slightly modified procedure reported by Swager and coworkers,[S3] a flat Erlenmeyer flask was filled with 2-nitrotriptycene (2.00 g, 6.68 mmol), 10% Pd/C (200 mg) and absolute EtOH (200 mL). After 3 vacuum/H_2 cycles, the mixture was kept stirring under hydrogen atmosphere at room temperature for 6 hours (during which time the progressive solubilization of the initial white solid was observed resulting in a black suspension). After monitoring the disappearance of the starting material by TLC (DCM/Hex 1/1), the mixture was filtered using celite and concentrated *in vacuo* obtaining 2-aminotripticene (1.73 g, 96%). Spectroscopic data were in agreement with the literature.

^{1}H NMR (200 MHz, d_6-DMSO) δ = 7.44–7.26 (m, 4H), 7.03 (d, J = 7.9 Hz, 1H), 6.95 m, 4H), 6.69 (d, J = 2.2 Hz, 1H), 6.12 (dd, J = 7.8, 2.2 Hz, 1H), 5.37 (s, 2H), 4.88 (s, 2H).

Route B

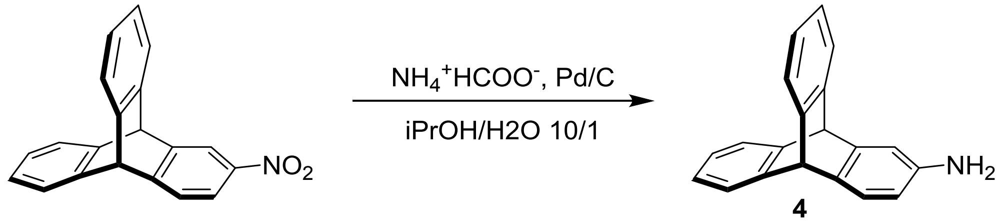

A mixture of isopropanol (10 mL), Pd/C 10% (10 mg) and ammonium formate (210 mg, 3.34 mmol, 10 eq) dissolved in H_2O (1mL) were stirred for 10 min to activate Pd/C. 2-Nitrotriptycene (100 mg, 0.334 mmol, 1 eq) was then added. The reaction was monitored by Thin Layer Chromatography (TLC, DCM/Hex 1/1 as eluent mixture). After completion of the reaction, the mixture was filtered on celite and concentrated *in vacuo*. Subsequently, the solid was dissolved in AcOEt and washed with brine. The organic phase was collected and dried with Na_2SO_4, and then concentrated *in vacuo* yielding 2-aminotripticene (60 mg, 67%). Spectroscopic data in agreement with reports in literature.[S3]
^{1}H NMR (200 MHz, d_6-DMSO) δ 7.44 – 7.26 (m, 4H), 7.03 (d, J = 7.9 Hz, 1H), 6.95 m, 4H), 6.69 (d, J = 2.2 Hz, 1H), 6.12 (dd, J = 7.8, 2.2 Hz, 1H), 5.37 (s, 2H), 4.88 (s, 2H).

Compound 11

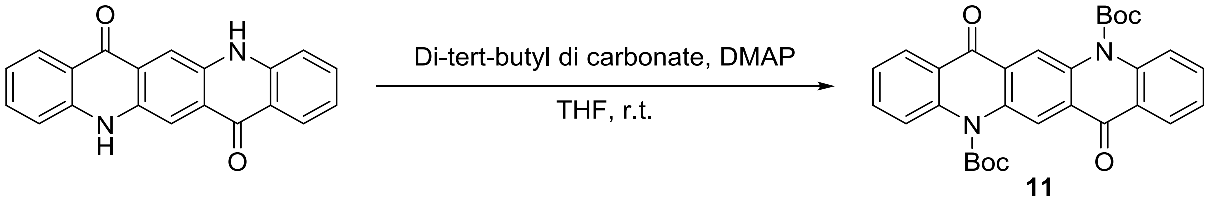

A solution of di-*tert*-butyl dicarbonate (0.47 g, 2.15 mmol, 6.70 eq) dissolved in anhydrous THF (1 mL) was added slowly to a suspension of commercial quinacridone (100 mg, 0.320 mmol, 1 eq), DMAP (235 mg, 1.92 mmol, 6 eq) in anhydrous THF (6 mL) at room temperature and under N_2 protection. The reaction was kept at room temperature for overnight. The yellow solution was then poured into water and extracted with DCM. The organic layers were combined and dried with anhydrous Na_2SO_4. After removing the solvent under reduced pressure, the residue was purified by column chromatography (SiO_2, DCM/hexane 4/1) to obtain **11** as a yellow powder (100 mg, 60%).
^{1}H NMR (200 MHz, $CDCl_3$) δ 8.75 (s, 2H), 8.42 (dd, J = 8.3, 2.0 Hz, 2H), 7.85 (d, J = 8.6 Hz, 2H), 7.78 – 7.63 (t, J = 8.3 Hz, 2H), 7.40 (t, J = 7.6, Hz, 2H), 1.77 (s, 18H).

Compound 12

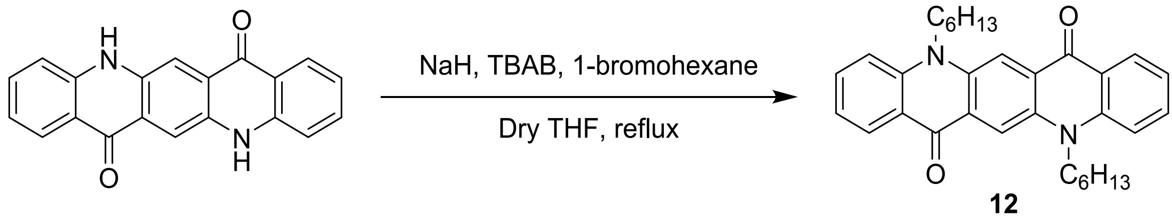

Commercial quinacridone pigment was alkylated according to literature.[S4] Sodium hydride (77 mg, 1.92 mmol, 6 eq) and tetrabutylammonium bromide (TBAB) (354 mg, 0.960 mmol, 3 eq) were added to a three-necked bottom round flask containing a suspension of quinacridone (100 mg, 1 eqv, 0.320 mmol) in dry THF (10 mL). The mixture was heated to reflux using an oil bath and stirred for 1 h, then 1-bromohexane (270 uL, 1.92 mmol, 6 eq) was added at once under nitrogen atmosphere. The mixture was kept refluxing for 24 hours. After that time, methanol (20 mL) was slowly added dropwise to the stirred mixture solution to decompose excess sodium hydride. The unreacted starting material was filtered off and the solvent was removed *in vacuo*. The residual crude solid product was further purified by column chromatography (SiO_2, petroleum ether/DCM 1/9) to obtain pure **12** as a red solid (116 mg, yield 75%). Spectroscopic data were in agreement with reports in the literature. ^{1}H NMR (200 MHz, $CDCl_3$) δ 8.80 (s, 2H), 8.59 (dd, *J* = 8.0, 1.7 Hz, 2H), 7.78 (dd, *J* = 8.7, 6.8, Hz, 2H), 7.53 (d, *J* = 8.7 Hz, 2H), 7.41 – 7.07 (m, 2H), 4.53 (t, *J* = 8.2 Hz, 4H), 2.01 (q, *J* = 7.8 Hz, 4H), 1.74 – 1.22 (m, 12H), 1.08 – 0.84 (m, 3H).

Synthesis of new compounds

Compound 7

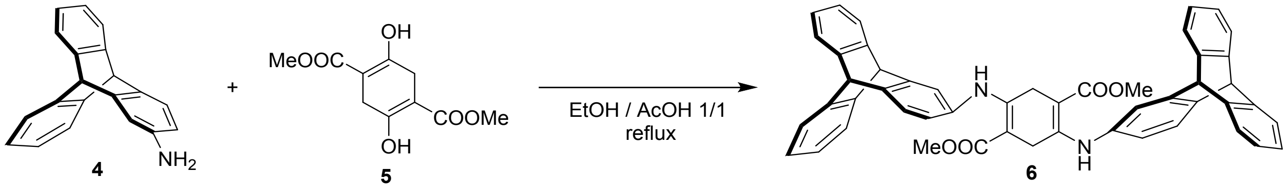

A 2-necked round bottom flask was filled with 2-aminotripticene (150 mg, 0.557 mmol, 4 eq), dimethyl succinyl succinate **5** (32 mg, 0.139 mmol, 1 eq), AcOH (0.500 mL) and EtOH (0.500 mL) under nitrogen atmosphere. The reaction mixture was heated to under reflux using an oil bath and stirred for 3 h, during which time the precipitation of an orange solid was observed. After checking the evolution of the reaction by TLC (Hex/AcOEt 7/3), the mixture was cooled to room temperature and filtered over Buchner, and the filtrate washed with EtOH. A fluorescent bright-orange solid **6** (98 mg, 96%) was obtained and used for the next step without any purification or characterization.

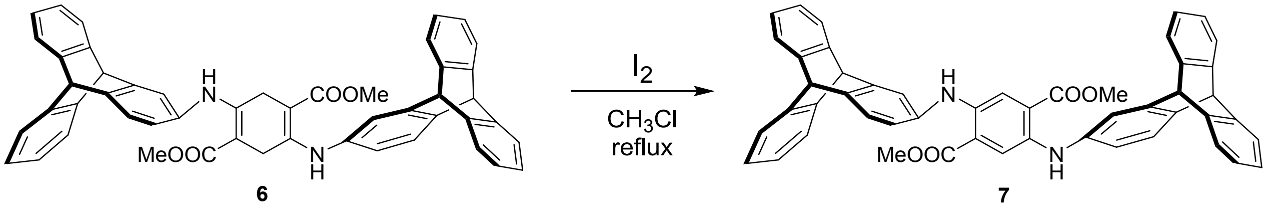

A 2-necked round bottom flask was filled with **6** (102 mg, 0.139 mmol, 1 eq), I_2 (36 mg, 0.139 mmol, 1 eq), CH_3Cl (3mL) and EtOH (1 mL). The solution was then stirred and heated to reflux at 80 °C using an oil bath overnight. After monitoring the evolution of the reaction by TLC (DCM/MeOH 95/5), the resulting deep red mixture was cooled to room temperature and concentrated *in vacuo*. The precipitate formed was suspended in ethanol and filtered affording pure product **7** (80 mg, 80%) as a bright orange solid. ^1H NMR (300 MHz, DMSO) δ 8.41 (s, 2H), 7.69 (s, 2H), 7.43 (dd, *J* = 5.4, 3.2 Hz, 8H), 7.34 (d, *J* = 7.9 Hz, 2H), 7.23 (d, *J* = 2.2 Hz, 2H), 7.00 (dd, *J* = 5.5, 3.1 Hz, 8H), 6.74 (dd, *J* = 7.9, 2.2 Hz, 2H), 5.57 (s, 2H), 3.70 (s, 2H).-^{13}C NMR (75 MHz, DMSO) δ 166.72, 146.74, 145.55, 145.13, 139.15, 138.80, 137.00, 124.99, 124.85, 124.23, 123.59, 123.41, 120.01, 119.11, 115.68, 114.66, 52.64, 52.32, 51.89. ESI (*m/z*): 729.33 $[M + 1]^+$ (100). Anal. Calcd. for $C_{50}H_{36}N_2O_4$, C, 82.4; H, 5.0; Found: C, 82.3; H, 5.0.

Compound 8

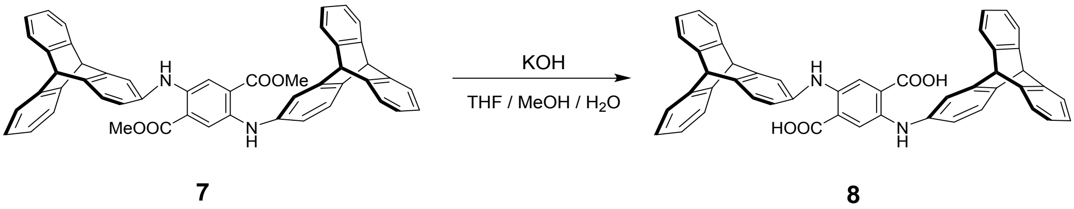

A two-necked round bottom flask was filled with **7** (120 mg, 0.165 mmol, 1 eq), KOH (9 mg, 0.165 mmol, 1 eq), MeOH (5 mL), THF (10 mL) and H_2O (10 mL). The mixture was stirred and heated to reflux at 80 °C using an oil bath overnight under Argon. The mixture was then cooled to room temperature and poured into H_2O. Subsequently, this solution was acidified to pH 2 with 2M HCl, yielding a precipitate that was filtered over a Buchner filter. Compound **8** (115 mg, 98%) was obtained as a red-purple solid. ^1H NMR (300 MHz, DMSO) δ 13.35 (s, broad, 2H), 8.73 (s, 2H), 7.73 (s, 2H), 7.42 (td, *J* = 7.3, 2.5 Hz, 8H), 7.34 (d, *J* = 8.0 Hz, 2H), 7.23 (d, *J* = 2.2 Hz, 2H), 7.16 – 6.89 (m, 8H), 6.76 (dd, *J* = 7.9, 2.2 Hz, 2H), 5.57 (s, 2H), 5.56 (s, 2H). ^{13}C NMR (75 MHz, DMSO) δ 168.60, 146.78, 145.59, 145.15, 139.16, 138.74, 137.24, 125.00, 124.85, 124.23, 123.61, 123.42, 119.97, 118.88, 115.64, 114.60, 52.61, 51.90. ESI (*m/z*): 701.79 $[M+1]^+$. Anal. Calcd. for $C_{48}H_{32}N_2O_4$: C, 82.3; H, 4.6. Found C, 82.0; H, 4.9.

Compound 3

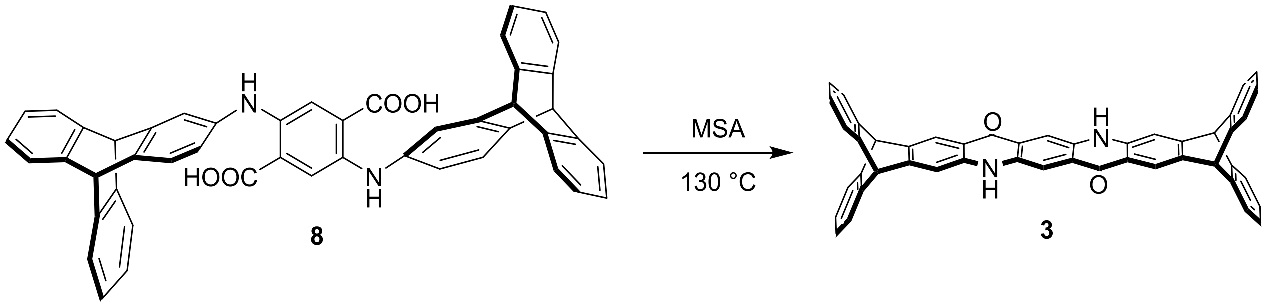

Compound **8** (400 mg, 0.570 mmol) was dissolved in methanesulfonic acid (MSA) (7 mL) and the reaction mixture was heated at 120 °C under nitrogen atmosphere for 5 h using an oil bath. The resulting deep purple solution was then poured into H_2O. The precipitate thus formed was filtered several times over Buchner until the aqueous phase was completely clear. After that the solid was washed with distilled H_2O and MeOH. Compound **3** (305 mg, 80%) was obtained as red/purple solid. A fraction used for analytical characterization was further purified by column chromatography (SiO_2, DCM/MeOH 98/2). ^{1}H NMR (400 MHz, *d*$_6$-DMSO) δ 11.76 (s, 2H), 8.40 (s, 2H), 8.18 (s, 2H), 7.55 (s, 2H), 7.50 (m, 8H), 7.28 – 6.89 (m, 8H), 5.85 (s, 2H), 5.80 (s, 2H). ^{13}C NMR (75 MHz, d$_6$-DMSO) δ 176.22, 150.76, 144.90, 143.73, 140.13, 137.22, 134.54, 125.57, 125.29, 124.65, 124.04, 123.67, 119.67, 115.70, 113.52, 112.18, 52.35, 51.55. ESI (*m/z*): 665.31 $[M + 1]^+$ (100). Anal. Calcd. for $C_{48}H_{28}N_2O_2$: C, 86.7; H, 4.3. Found: C, 86.8; H, 4.4.

Synthesis of compound 9

Route A:

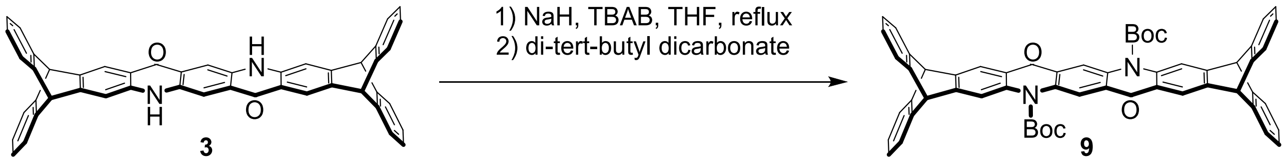

Triptycene quinacridone **3** (155 mg, 0.233 mmol, 1 eq) and tetrabutylammonium bromide (TBAB) (258 mg, 0.700 mmol, 3 eq) were dissolved in anhydrous THF (10 mL). NaH 60 % w/w (93 mg, 2.33 mmol, 10 eq) was added and the reaction mixture was heated to reflux. After 1 h, di-*tert*-butyl dicarbonate (509 mg, 2.33 mmol, 10 eq) dissolved in dry THF (1 mL) was added, and the mixture was kept at reflux for 24 h. After this time, methanol was added to the mixture in order to neutralize the excess NaH, and then solvents were removed *in vacuo*. The mixture was suspended in water and extracted with EtOAc. Organic phases were combined, dried over Na_2SO_4, filtered and the solvents

were removed *in vacuo*. This sample was purified by column chromatography (SiO_2, Hex/EtOAc 8/3) obtaining pure compound **9** as a yellow powder (17 mg, 8%).

Route B

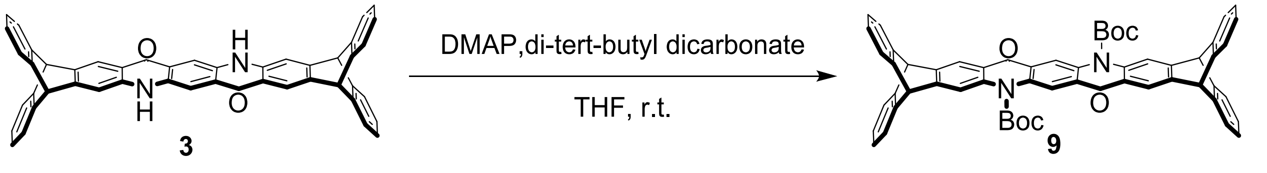

A solution of di-*tert*-butyl decarbonate (263 mg, 8.00 eqv, 1.20 mmol) in anhydrous THF (1.0 mL) was added slowly to a solution of compound **3** (100 mg, 1.00 eqv, 0.15 mmol) and DMAP (37 mg, 2.00, eqv g, 1.72 mmol) in anhydrous THF (6 mL) at room temperature under nitrogen atmosphere. The reaction mixture was kept at room temperature overnight. The yellow solution was then poured into H_2O and extracted with DCM. The organic layers were combined and dried with anhydrous Na_2SO_4. After removing the solvent in vacuo, the residue was purified by column chromatography (SiO_2, Hex/EtOAc 9/3) to obtain **9** as a yellow powder (30 mg, 23%). ^{1}H NMR (300 MHz, $CDCl_3$) δ 8.62 (s, 2H), 8.31 (s, 2H), 7.91 (s, 2H), 7.44 - 7.38 (m, 8H), 7.15 – 7.00 (m, 8H), 5.54 (s, 2H), 5.53 (s, 2H), 1.72 (s, 18H). ^{13}C NMR (75 MHz, $CDCl_3$) δ 178.32, 151.73, 151.52, 144.61, 143.62, 140.71, 138.69, 134.91, 126.35, 126.02, 125.76, 124.17, 123.96, 121.00, 120.25, 117.73, 114.49, 86.66, 54.60, 53.28, 28.03. DEP (*m/z*): 664.23 $[M]^{\cdot+}$ (100). Anal. Calcd. for $C_{58}H_{44}N_2O_6$: C, 80.5; H, 5.1. Found: C, 80.4; H, 5.0.

Compound 10

Route A

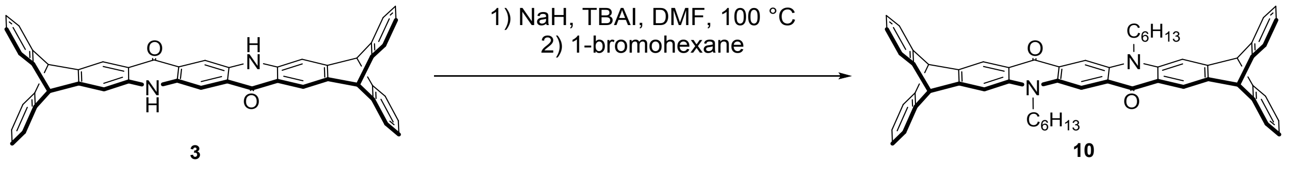

Triptycene-fused quinacridone **3** (50 mg, 0.075 mmol, 1 eq) and tetrabutylammonium iodide (TBAI) (83 mg, 0.226 mmol, 3 eq) were dissolved in anhydrous DMF (10 mL). NaH 60 % w/w (36 mg, 0.903 mmol, 12 eq) was added and the reaction mixture was heated to 100 °C. After 1 h, 1-bromohexane (127 uL, 0.903 mmol, 12 eq) was added to the reaction mixture, and the mixture was kept at 100 °C under stirring for 24 h. After this time, methanol was added to the mixture to neutralize the excess NaH and then the solvents were removed *in vacuo*. The solid was suspended in H_2O and extracted with EtOAc, the organic phases were dried over Na_2SO_4, filtered and the solvents removed under

reduced pressure. The solid was then purified by column chromatography (SiO_2, Hex/EtOAc 8/3) to obtain pure compound **10 as** a red solid (5 mg, 8%).

Route B

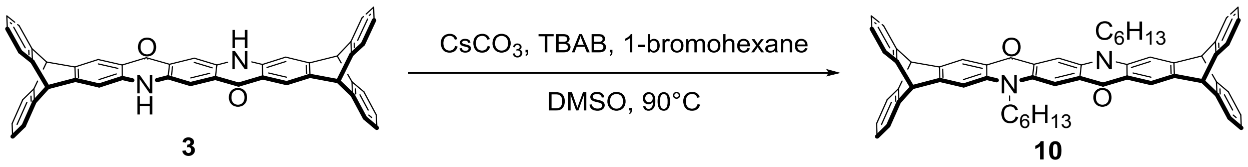

Compound **3** (50 mg, 0.075 mmol, 1 eq), cesium carbonate (49 mg, 0.150 mmol, 2 eq) and tetrabutylammonium bromide (TBAB) (5 mg, 0.015 mmol, 0.2 eq) were dissolved in DMSO (2 mL) and stirred at room temperature for 4 h. After that, 1-bromohexane (4.98 g, 20 mmol) was slowly added dropwise. The resulting mixture was allowed to react at room temperature for 20 h, and then the mixture was heated to 90 °C for 6 h. After cooling to room temperature, the reaction mixture was poured H_2O and filtered under reduced pressure. The resulting crude product was purified by column chromatography (SiO_2, Hex/AcOEt 9/3) to obtain compound **10** as a red solid (16 mg, 25%). ^1H NMR (400 MHz, $CDCl_3$) δ 8.82 (s, 2H), 8.54 (s, 2H), 7.58 (s, 2H), 7.55 – 7.36 (m, 8H), 7.17 – 6.79 (m, 8H), 5.61 (s, 2H), 5.59 (s, 2H), 4.52 (t, J = 8.7 Hz, 4H), 1.65 (q, J = 7.5 Hz, 4H), 1.60 – 1.30 (m, 14H), 0.97 (t, J = 7.0 Hz, 6H). ^{13}C NMR (75 MHz, DMSO) δ 177.58, 151.95, 144.94, 143.67, 141.18, 137.71, 135.77, 126.26, 126.08, 125.68, 124.06, 123.99, 121.77, 118.49, 113.55, 110.07, 54.87, 53.01, 46.69, 31.65, 31.07, 29.84, 27.32, 26.79, 22.83, 14.20. HR MALDI-TOF (*m/z*): 833.2431 [M + H]$^+$. Anal. Calcd. for $C_{60}H_{52}N_2O_2$: C, 86.5; H, 6.3. Found: C, 86.6; H, 6.4.

2. ^{1}H-NMR comparison of commercial quinacridone and 3

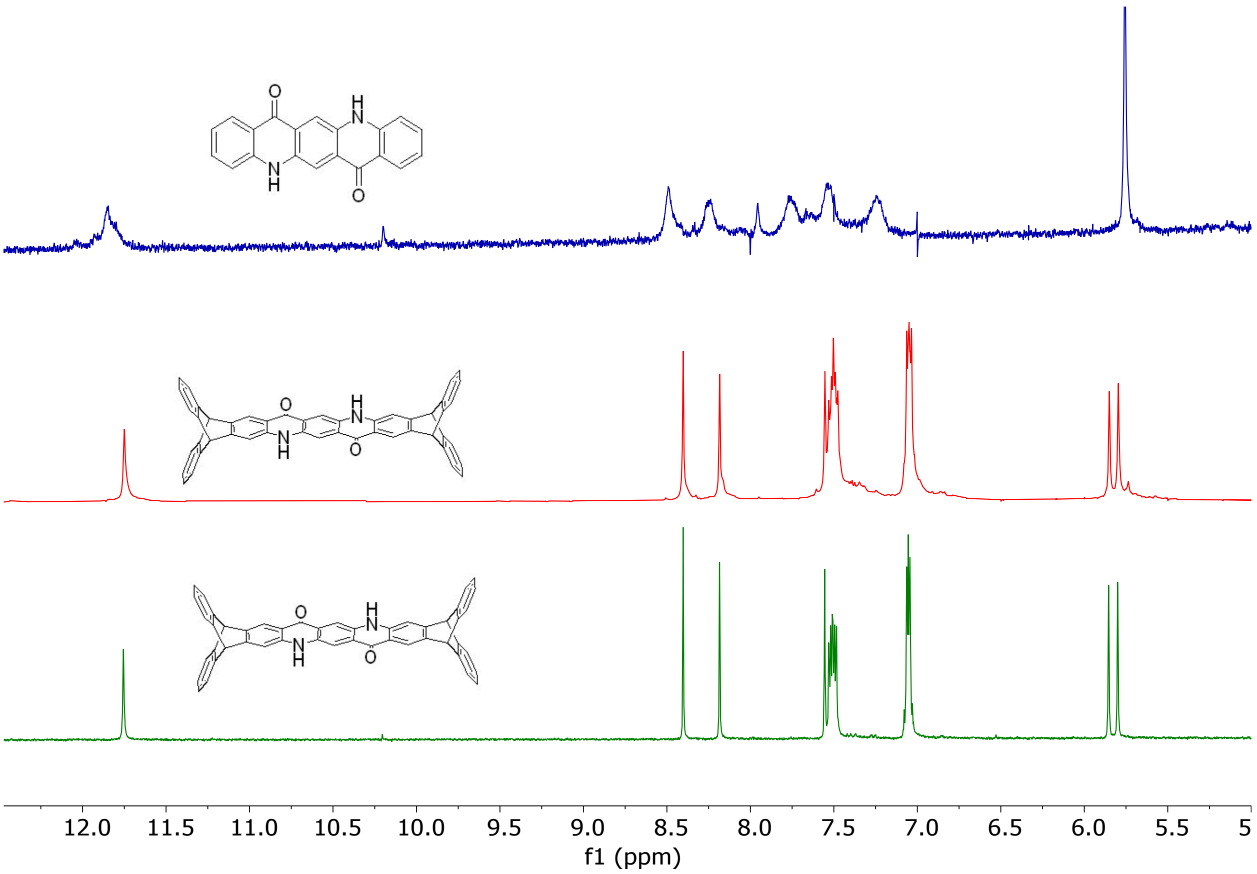

Figure S1. ^{1}H-NMR (300 MHz, DMSO-d_6) of commercial quinacridone pigment (blue spectrum), **3** after filtration (red spectrum) and **3** after column chromatography (SiO_2, DCM/MeOH 98/2).

3. UV-vis and emission studies

UV-Visible spectra were collected by Perkin-Elmer Lambda900 spectrophotometer. The solid UV-Vis diffuse reflectance spectra were recorded using a Shimadzu UV3600 spectrophotometer with $BaSO_4$ as a reference. Steady state emission and excitation spectra and photoluminescence lifetimes were obtained using both a FLS 980 (Edinburg Instrument Ltd) and a Nanolog (Horiba Scientific) spectrofluorimeter composed of iH320 spectrograph equipped with a Synapse QExtra charge-coupled device. The spectra are corrected for the instrument response. PL Quantum Yields of solutions are obtained by using Rhodamine 6G as the reference. PL QY of solid-state samples were measured with a home-made integrating sphere according to the procedure reported elsewhere.[S5] Time-resolved TCSPC measurements are obtained with PPD-850 single photon detector module and DeltaTime series DD-300 DeltaDiode and DD-405L DeltaDiode Laser and analyzed with the instrument Software DAS6. Decay fits are performed with multi-exponential functions and average lifetimes obtained as $\tau_{av} = \frac{\sum_{n=1}^{m} B_n t_n^2}{\sum_{n=1}^{m} B_n t_n}$.

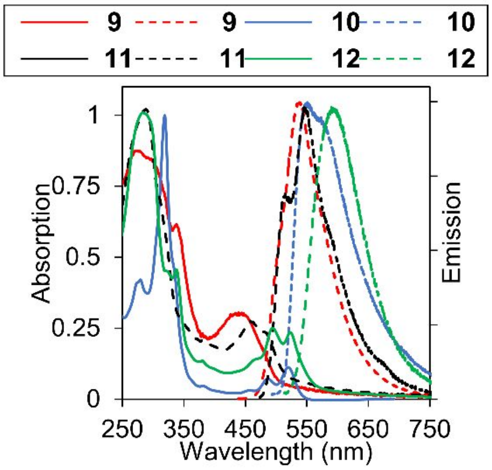

Figure S2. Absorption (solid lines) and emission (dotted lines) spectra in cast film from chloroform solution for **9**, **10**, **11** and **12**.

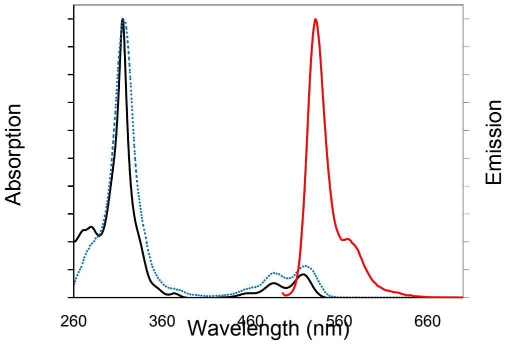

Figure S3. Absorption (black line), emission (λ_{exc}= 534 nm, red line) and PL excitation ((λ_{em}= 486 nm blue dashed line) spectra of compound **3** in DMSO.

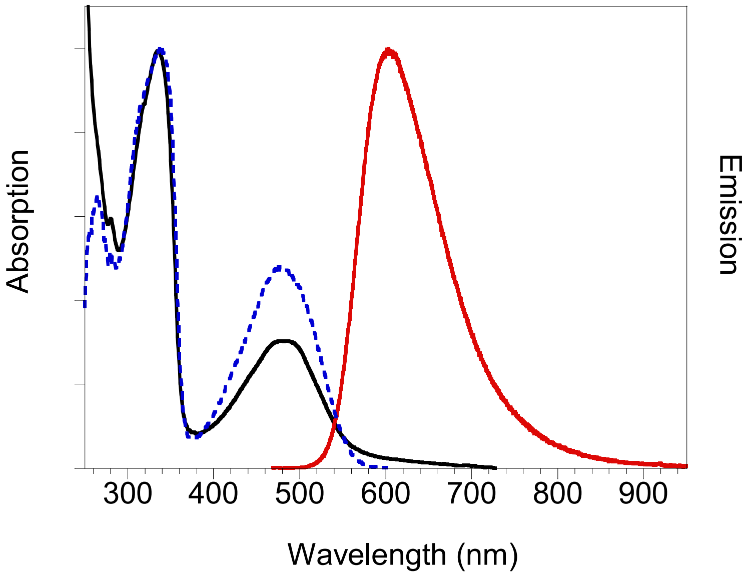

Figure S4. Absorption (black line), emission (λ_{exc}= 470 nm, red line) and excitation (λ_{em}= 605 nm, blue line) spectrum of **7** in chloroform solution.

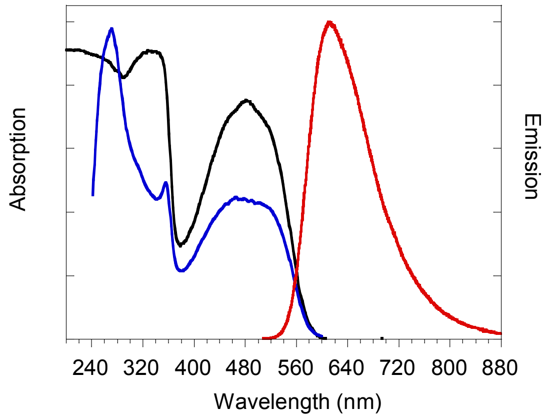

Figure S5. Absorption (black line), PL excitation (λ_{em}= 612 nm, blue line) and emission (λ_{exc}= 420 nm, red line) of **7** in cast film from chloroform solution.

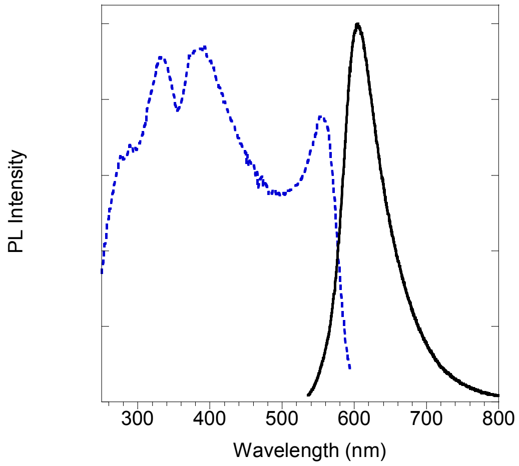

Figure S6. PL excitation (λ_{em}= 605 nm, blue dashed line) and emission (λ_{exc}= 450 nm, black line) of **7** from powder.

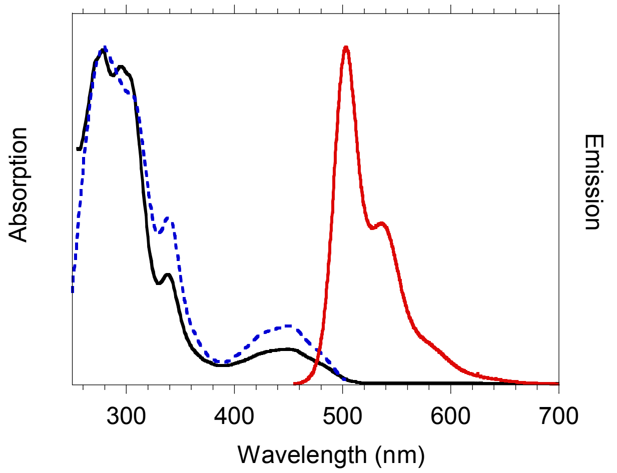

Figure S7. Absorption (black line), PL excitation (λ_{em}= 505 nm, blue dashed line) and emission (λ_{exc}= 443 nm, red line) spectra of **9** in chloroform solution.

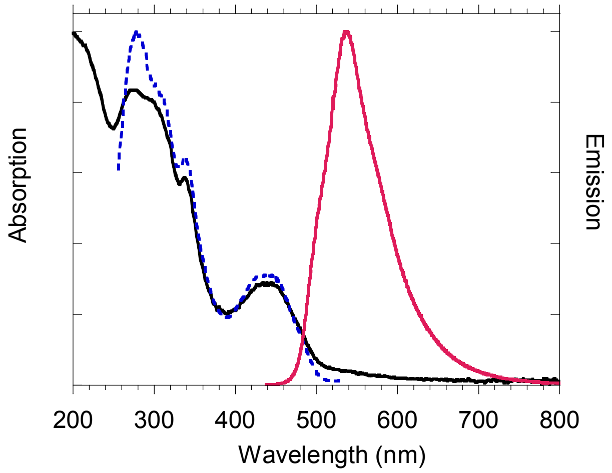

Figure S8. Absorption (black line), PL excitation (λ_{em}= 537 nm, blue line) and emission (λ_{exc}= 420 nm, red line) of **9** in cast film from chloroform solution.

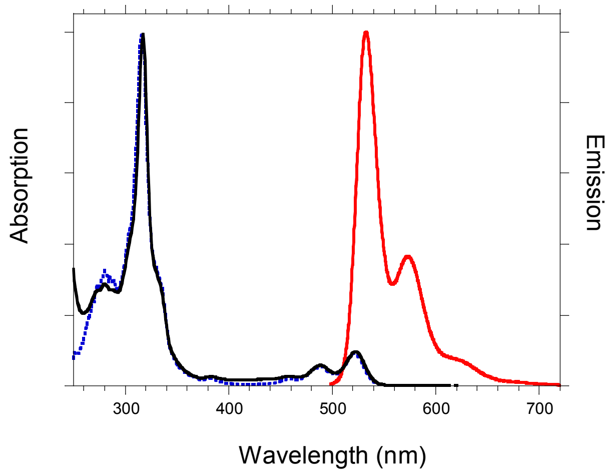

Figure S9. Absorption (black line), PL excitation (λ_{em}= 575 nm, blue dashed line) and emission (λ_{exc}= 317 nm, red line) spectrum of **10** in chloroform solution.

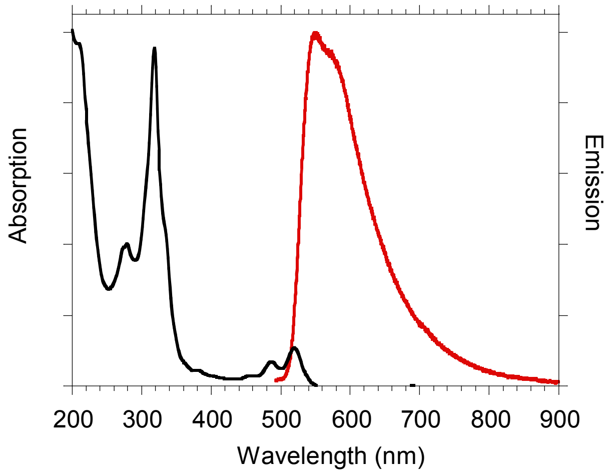

Figure S10. Absorption (black line) and emission (λ_{exc}= 488 nm, red line) of **10** in cast film from chloroform solution.

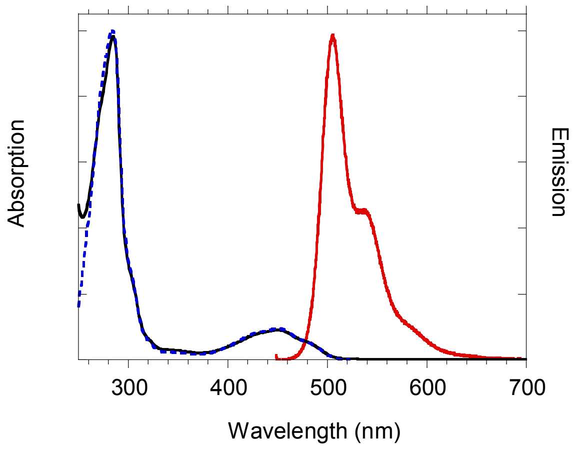

Figure S11. Absorption (black line), PL excitation (λ_{em}= 538 nm, blue dashed line) and emission (λ_{exc}= 450 nm, red line) spectrum of **11** in chloroform solution.

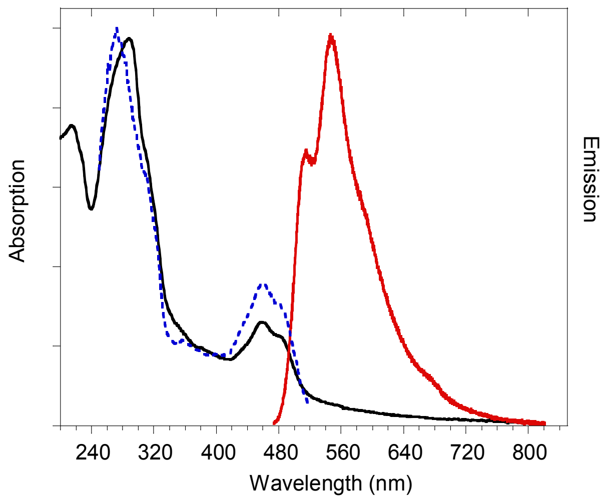

Figure S12. Absorption (black line), PL excitation (λ_{em}= 550 nm, blue dashed line) and emission (λ_{exc}= 460 nm, red line) of **11** in cast film from chloroform solution.

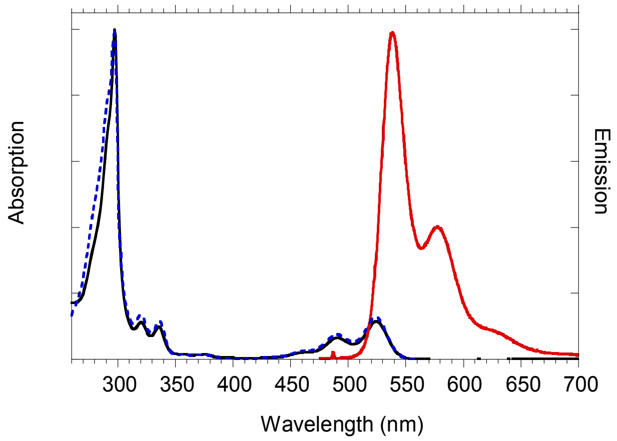

Figure S13. Absorption (black line), PL excitation (λ_{em}= 578 nm, blue dashed line) and emission (λ_{exc}= 488 nm, red line) spectrum of **12** in chloroform solution.

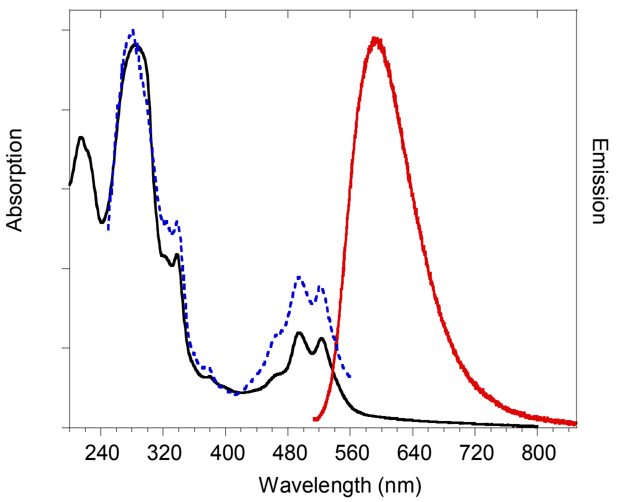

Figure S14. Absorption (black line) PL excitation (λ_{em}= 592 nm, blue dashed line) and emission (λ_{exc}= 490 nm, red line) of **12** in cast film from chloroform solution.

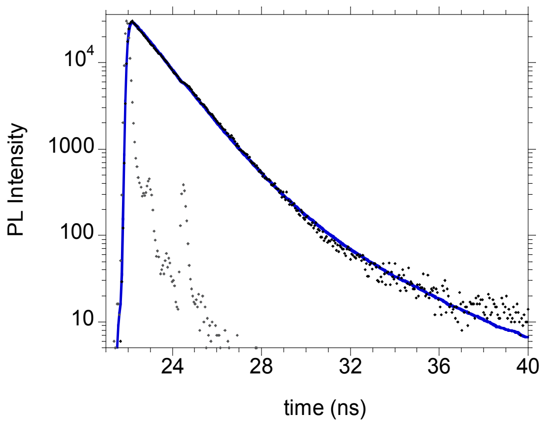

Figure S15. Photoluminescence decays of **7** chloroform solution (λ_{exc}= 408 nm, λ_{em}= 605 nm, black points) Prompt (grey points) and bi-exponential best fit (blue line). τ_{av}=1.37 ns; χ^2 = 3.021198; T_1 = 1.226016x10^{-9} s ; T_2 = 3.007861x10^{-9} sec; B_1 = 0.263855 [91.79 Rel.Ampl]; B_2 = 9.616138x10^{-3} [8.21 Rel.Ampl].

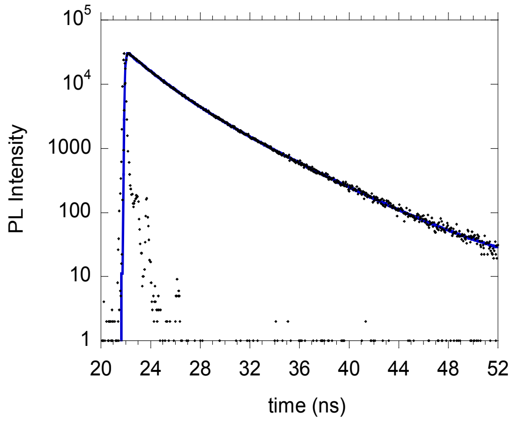

Figure S16. Photoluminescence decays of **7** as powders (λ_{exc}= 408 nm, λ_{em}= 590 nm, black points) Prompt (grey points) and bi-exponential best fit (blue line, τ_{av}=3.45 ns; χ^2 =2.894657; T_1 =2.148682x10^{-9} s; T_2 = 4.636804x10^{-9} s; B_1 = 0.2214526 [47.68 Rel.Ampl]; B_2 = 0.112601 [52.32 Rel.Ampl]).

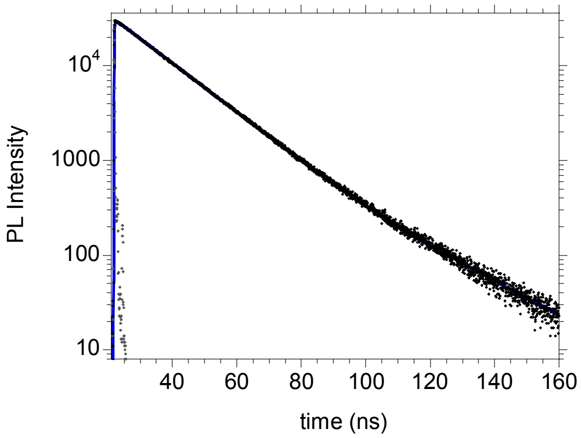

Figure S17. Photoluminescence decays of **9** chloroform solution (λ_{exc}= 408 nm, λ_{em}= 505 nm, black points) Prompt (grey points) and mono-exponential best fit (blue line, τ=16.76 ns; χ^2= 1.40519).

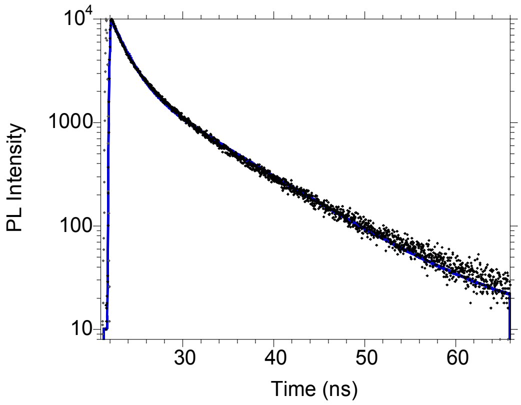

Figure S18. Photoluminescence decays of **9** cast film from chloroform solution (λ_{exc}= 408 nm, λ_{em}= 537 nm, black points) Prompt (grey points) and bi-exponential best fit (blue line, τ_{av}=5.62 ns; χ^2= 2.345933; T_1 = 1.71267x10^{-9} s; T_2 = 8.044704x10^{-9} s; B_1 = 0.1053188 [38.30 Rel.Ampl]; B_2 = 3.612159x10^{-2} [61.70 Rel.Ampl]).

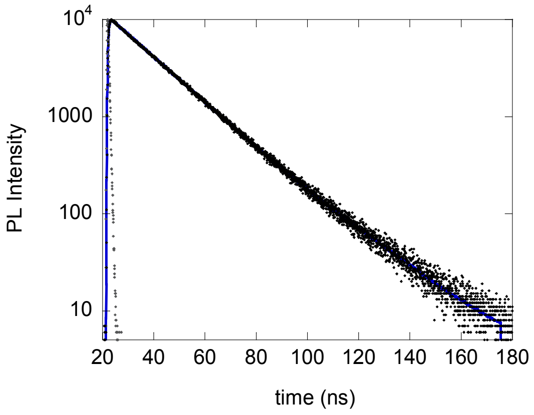

Figure S19. Photoluminescence decays of **10** chloroform solution (λ_{exc}= 300 nm, λ_{em}= 460 nm, black points) Prompt (grey points) and mono-exponential best fit (blue line, τ= 18.59 ns; χ^2= 1.076202).

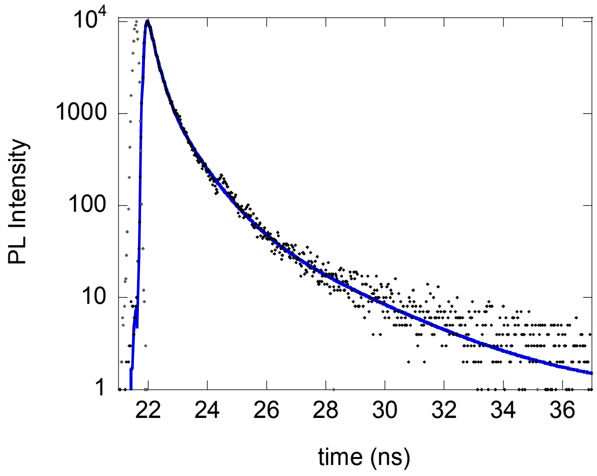

Figure S20. Photoluminescence decays of **10** film (λ_{exc}= 408 nm, λ_{em}= 570 nm, black points) Prompt (grey points) and three-exponential best fit (blue line, τ_{av}=580ps; χ^2 = 1.604737; T_1 = 2.572411x10^{-10} s; T_2 = 8.178286x10^{-10} s; T_3 = 2.739852x10^{-9} s; B_1 = 0.2511821; [62.62 Rel.Ampl]; B_2 = 3.968265x10^{-2}; [31.45 Rel.Ampl]; B_3 = 2.232312x10^{-3} [5.93 Rel.Ampl].

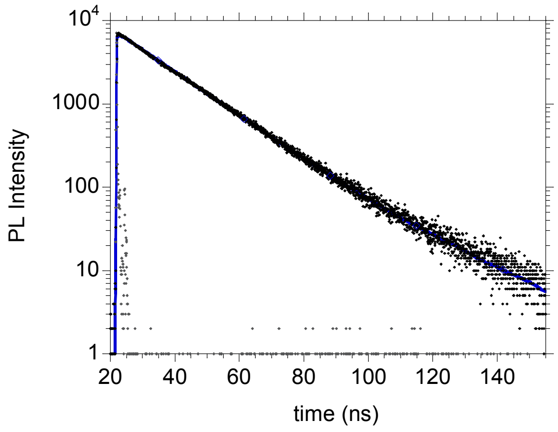

Figure S21. Photoluminescence decays of **11** chloroform solution (λ_{exc}= 408 nm, λ_{em}= 506 nm, black points) Prompt (grey points) and mono-exponential best fit (blue line, τ=16.46ns; χ^2 = 1.264374)

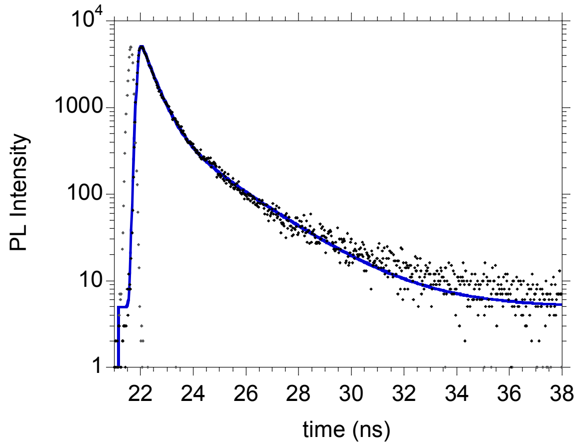

Figure S22. Photoluminescence decays of **11** cast film from chloroform solution (λ_{exc}= 408 nm, λ_{em}= 550 nm, black points) Prompt (grey points) and bi-exponential best fit (blue line). (blue line, τ_{av}=0.998 ns; T_1 = 4.759294x10^{-10} s; T_2 = 2.076048x10^{-9} s; B_1 = 0.1680685 [67.35 Rel.Ampl]; B_2 = 1.868055x10^{-2} [32.65 Rel.Ampl] χ^2 = 2.219152).

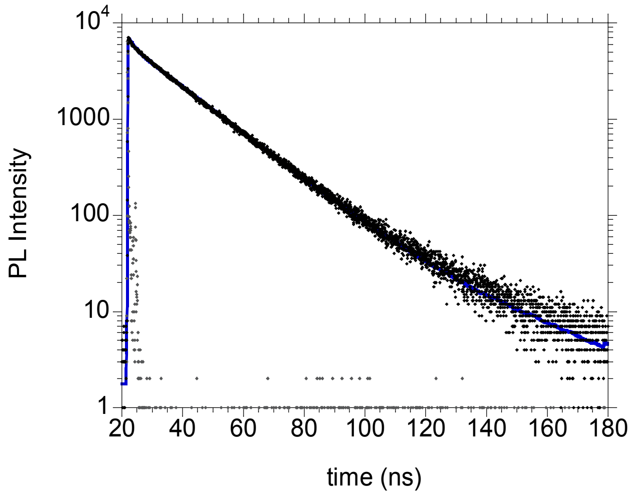

Figure S23. Photoluminescence decays of **12** from chloroform solution (λ_{exc}= 408 nm, λ_{em}= 538 nm, black points) Prompt (grey points) and bi-exponential best fit (blue line, τ_{av}=17.51ns; T_1 = 1.629214x10^{-9} s; T_2 = 1.800314x10^{-8} s. B_1 = 6.658726x10^{-2} [3.00 Rel.Ampl]; B_2 = 0.1946152 [97.00 Rel.Ampl] χ^2 = 1.200255).

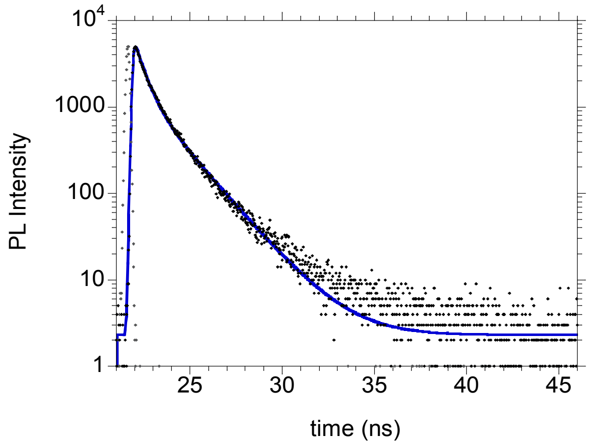

Figure S24. Photoluminescence decays of **12** cast film from chloroform solution (λ_{exc}= 408 nm, λ_{em}= 592 nm, black points) Prompt (grey points) and bi-exponential best fit (blue line, τ_{av}=1.192ns; T_1 = 5.138636x10^{-10} s; T_2 = 1.774687x10^{-9} s. B_1 = 0.1277333 [46.25 Rel.Ampl]; B_2 = 4.297797x10^{-2} [53.75 Rel.Ampl] χ^2 = 2.31638).

4. Cyclic voltammetry

Experiments were carried out using an Epsilon-Eclipse potentiostat with a polished glassy carbon working electrode, platinum counter electrode, silver pseudo-reference electrode, and tetrabutylammonium hexofluorophosphate (recrystallized three times from EtOH) as a supporting electrolyte. Sample concentrations were between 0.2 and 1.0 mM in CH_2Cl_2. All electrochemical measurements were referenced to the Fc/Fc^+ redox couple. Band gaps were estimated using the onset of the initial oxidation and reduction events, and E_{HOMO} and E_{LUMO} were estimated given an E_{HOMO} of 4.80 eV for ferrocene. In all figures the first scan is shown, which is similar to subsequent scans.

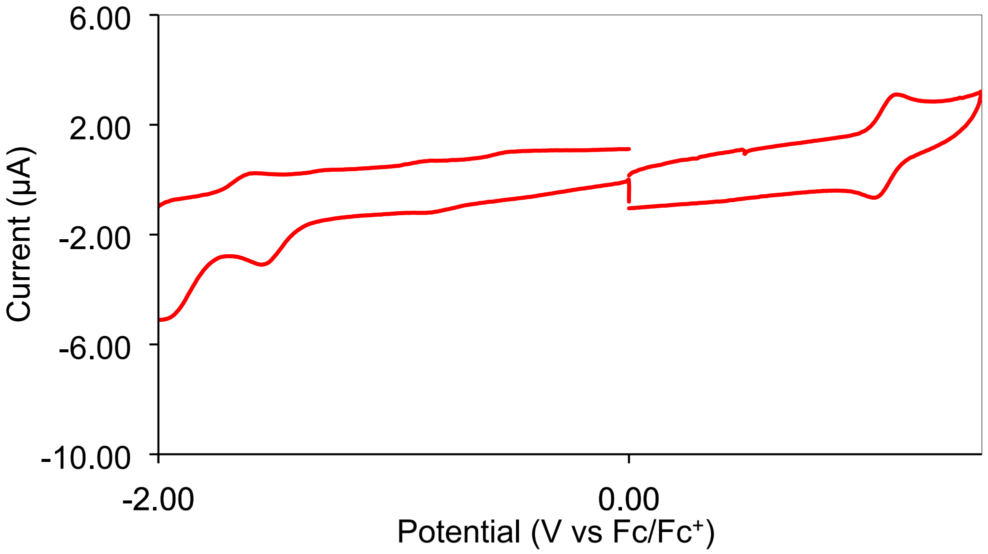

Figure S25. Cyclic voltammogram for compound **10**.

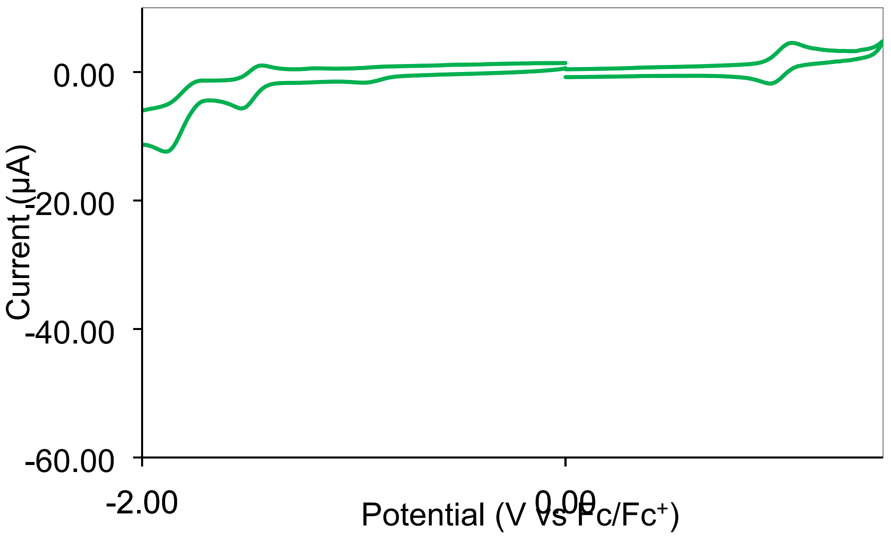

Figure S26. Cyclic voltammogram for compound **12**.

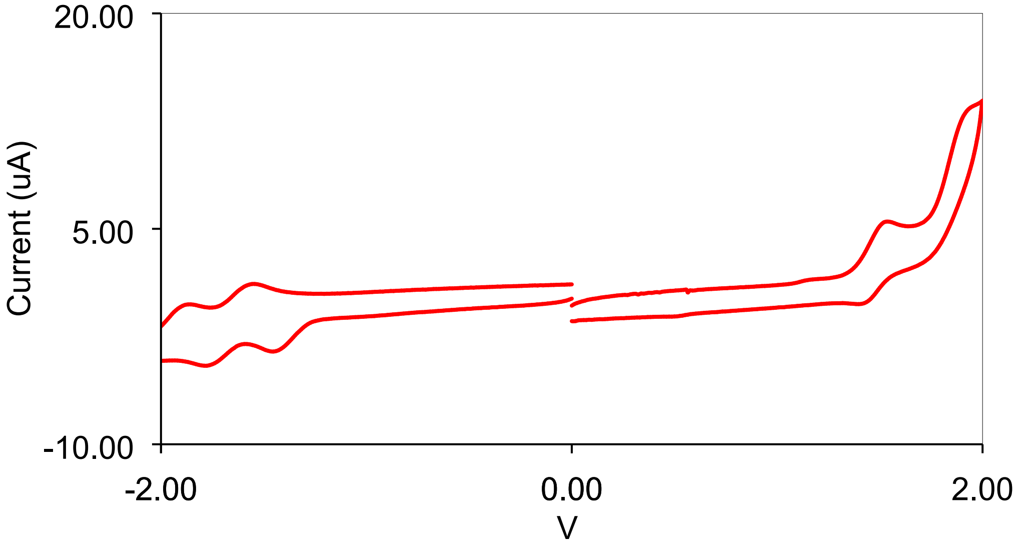

Figure S27. Cyclic voltammogram for compound **9**.

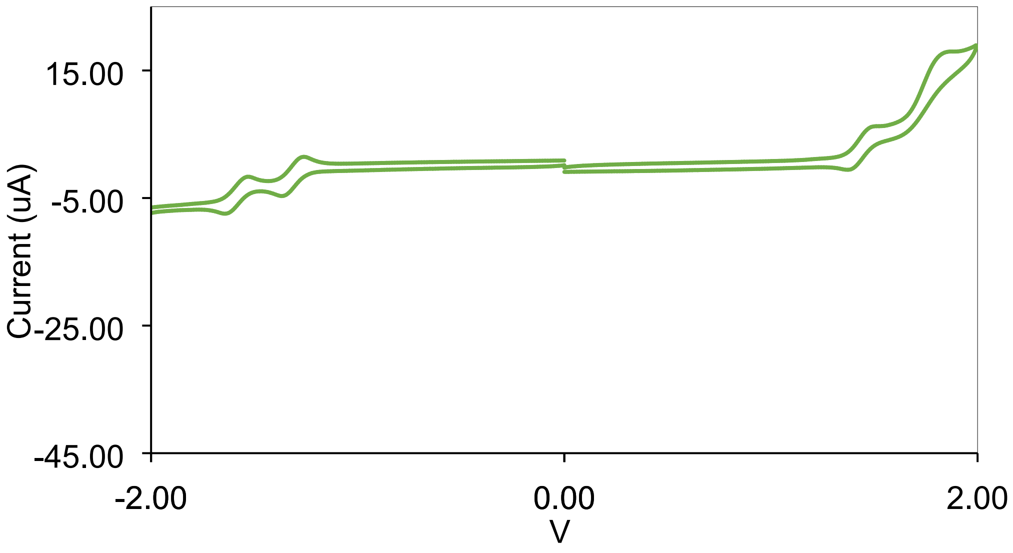

Figure S28. Cyclic voltammogram for compound **11**.

5. TGA of derivative 9

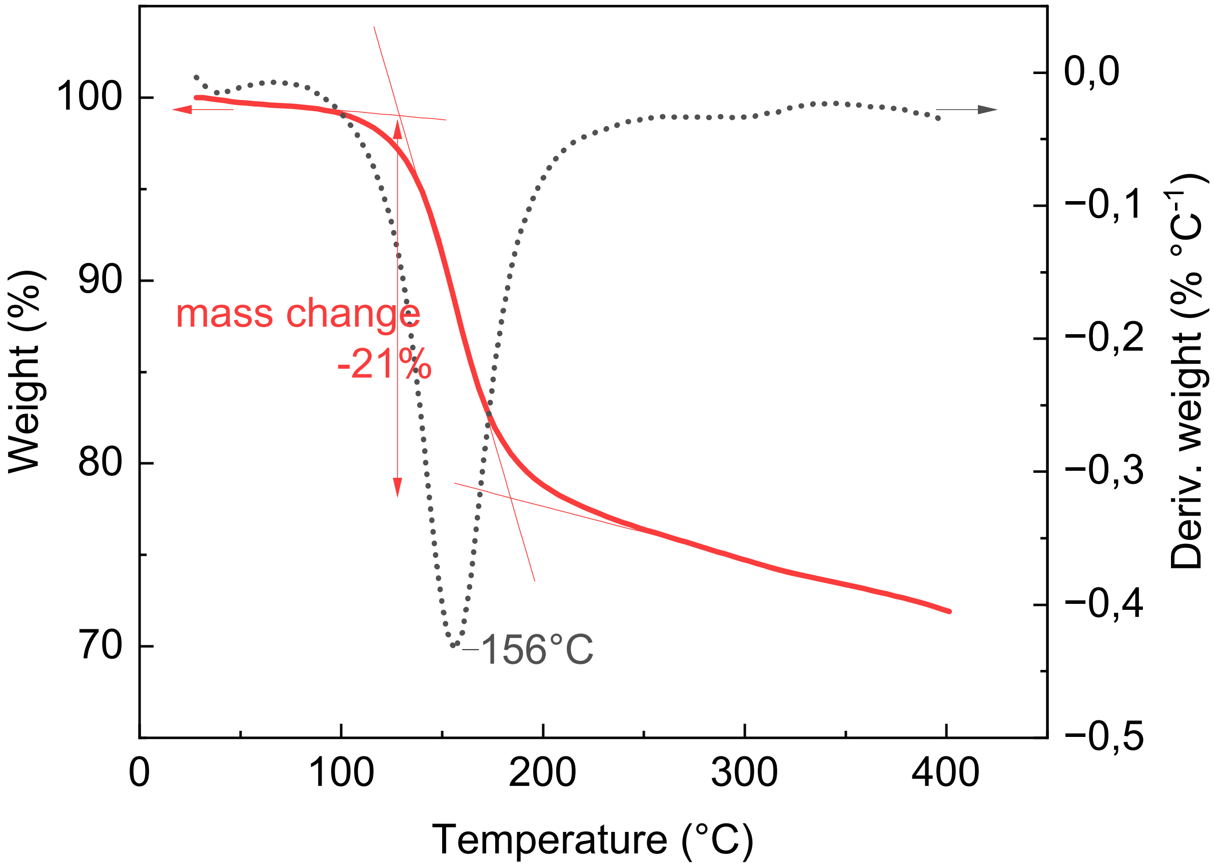

Figure S29. Thermogravimetric analysis of derivative **9** was performed on a TGA/DSC STARe System (Mettler Toledo) between 30 °C and 400 °C. The measurement was performed under N_2 atmosphere, and at a scanning speed of 10 °C min^{-1}. The observed mass loss (-21%) is consistent with the thermal cleavage of the Boc protecting groups, which account for 23% of the total mass of the derivative. The deprotection temperature (156 °C) is close to that reported for derivative **11** (175 °C).

6. Characterization in PMMA slabs

General procedure for PMMA slabs preparation. In a typical procedure for the preparation of a slab, AIBN (30 mg) was dissolved in freshly distilled methyl methacrylate (MMA) (100 mL). The solution was placed in an Erlenmeyer flask and slowly heated on a hot plate until the temperature of 100 °C was reached. This temperature was maintained for 5 min, during which time viscosity increased substantially. The solution was then immediately transferred in an ice bath and cooled at 20 °C. Compound **9** (15 mg) and lauryl peroxide (26 mg) were dissolved in 40 mL of the obtained MMA viscous syrup. The solution thus obtained was degassed for 30 min, and subsequently poured in a mold of 3 mm thickness and 100 cm^2 area and heated in a water bath at 58 °C for 24 h. At the end of this thermal treatment the syrup turns into a solid slab that is further thermally annealed.

Slab A: curing performed at 58 °C for 28 h.

Slab B: curing performed at 100 °C for 72 h.

After cooling, the slab can be easily separated from the glass mold and polished for optical measurements. In order to perform optical characterization, each slab was cut into pieces (dimensions 1x2 cm^2), and absorption spectrum was collected for each piece before and after treatment.

Slab A – cleavage of Boc protecting group by thermal treatment.[S6] Since the slab was cured at low temperature, derivative **9** still bears the Boc protecting groups at the end of the slab preparation. We used this sample to demonstrate thermal cleavage by treatment at higher temperature.

Each slab piece was heated at 100 °C in a closed vial on a DrySyn heating block for the appropriate amount of time, and subsequently the absorption spectrum was collected. Figure S30 shows the variation of the absorption spectrum of the samples over time. After 66 hours, the spectra of the samples stop changing, as Boc cleavage is complete, and derivative **3** is formed.

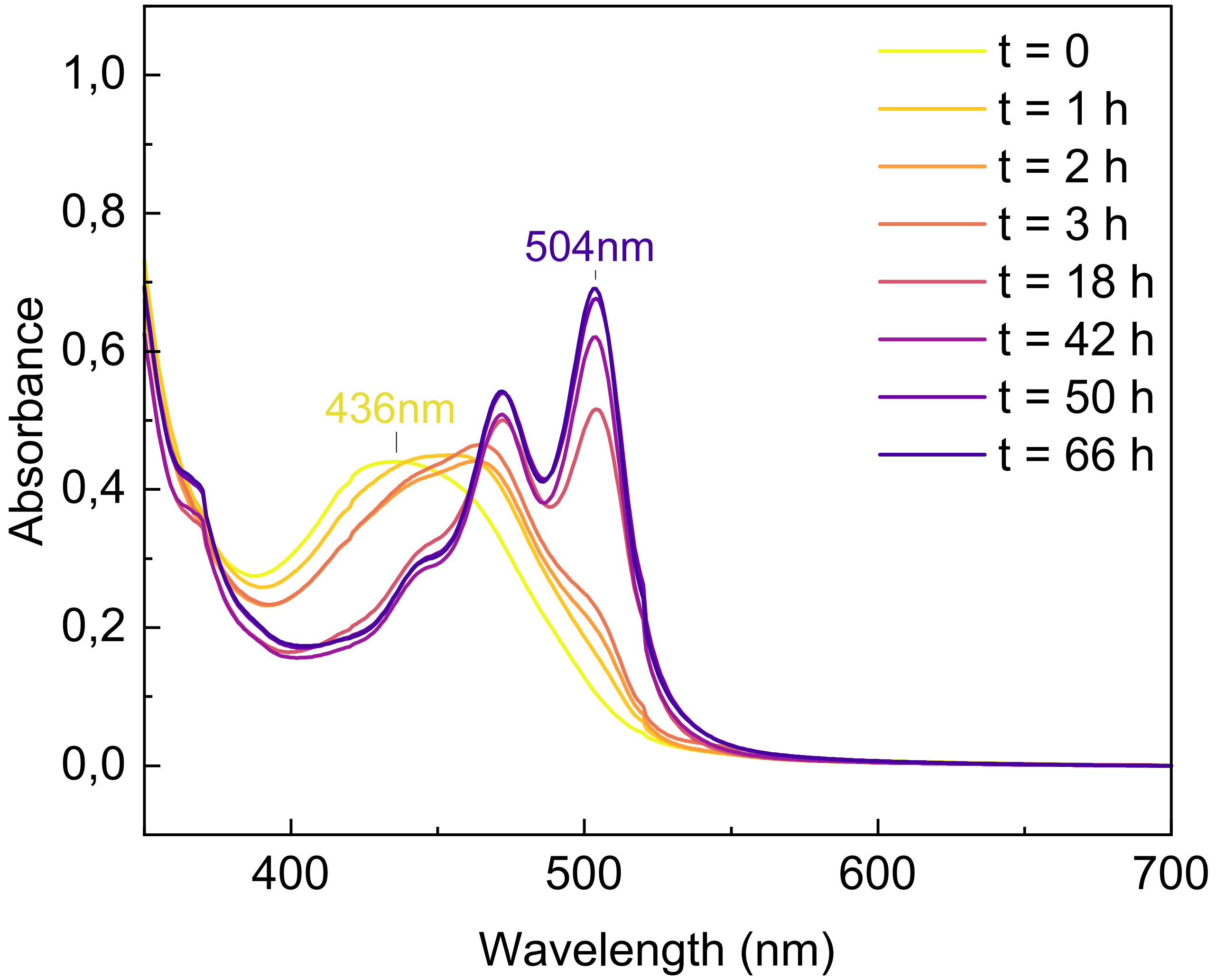

Figure S30. Absorption spectra as a function of treatment time at 100 °C of a PMMA slab containing **9**.

Slab B – stability of derivative 3 in PMMA slab. Since the slab was cured at 100 °C for 72 hours, derivative **9** is completely deprotected, thus **3** is present in the slab. We used this sample to study the stability of **3** under light irradiation.

Aging of samples was carried on in a Solarbox 3000 E at 80 °C and at an irradiation power of 2 Suns. After ageing, each spectrum was normalized on the basis of the absorbance of the corresponding pristine (non-irradiated) sample. Figure S31 shows the variation of the absorption spectrum of the samples over time. As a comparison, Figure S31 shows the shows the variation of the absorption spectrum of analogous PMMA samples containing quinacridone (**QA**), alkylated quinacridone (**Q-Alk**), and quinacridone in the presence of bis(1-octyloxy-2,2,6,6-tetramethyl-4-piperidyl) sebacate (**QA + stabilizer**) as the function of irradiation exposure time.

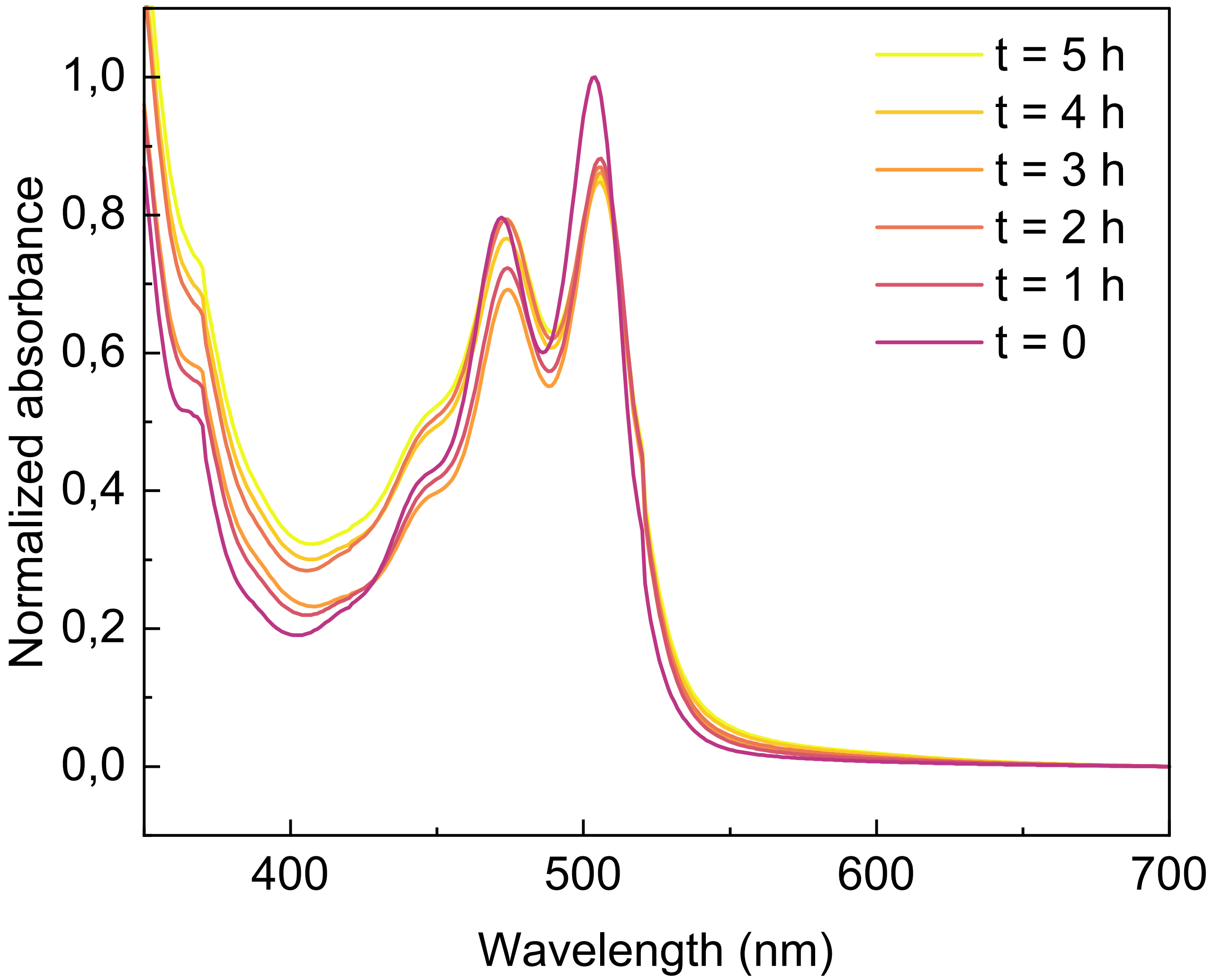

Figure S31. Absorption spectra as a function of irradiation exposure time of a PMMA slab containing **3**.

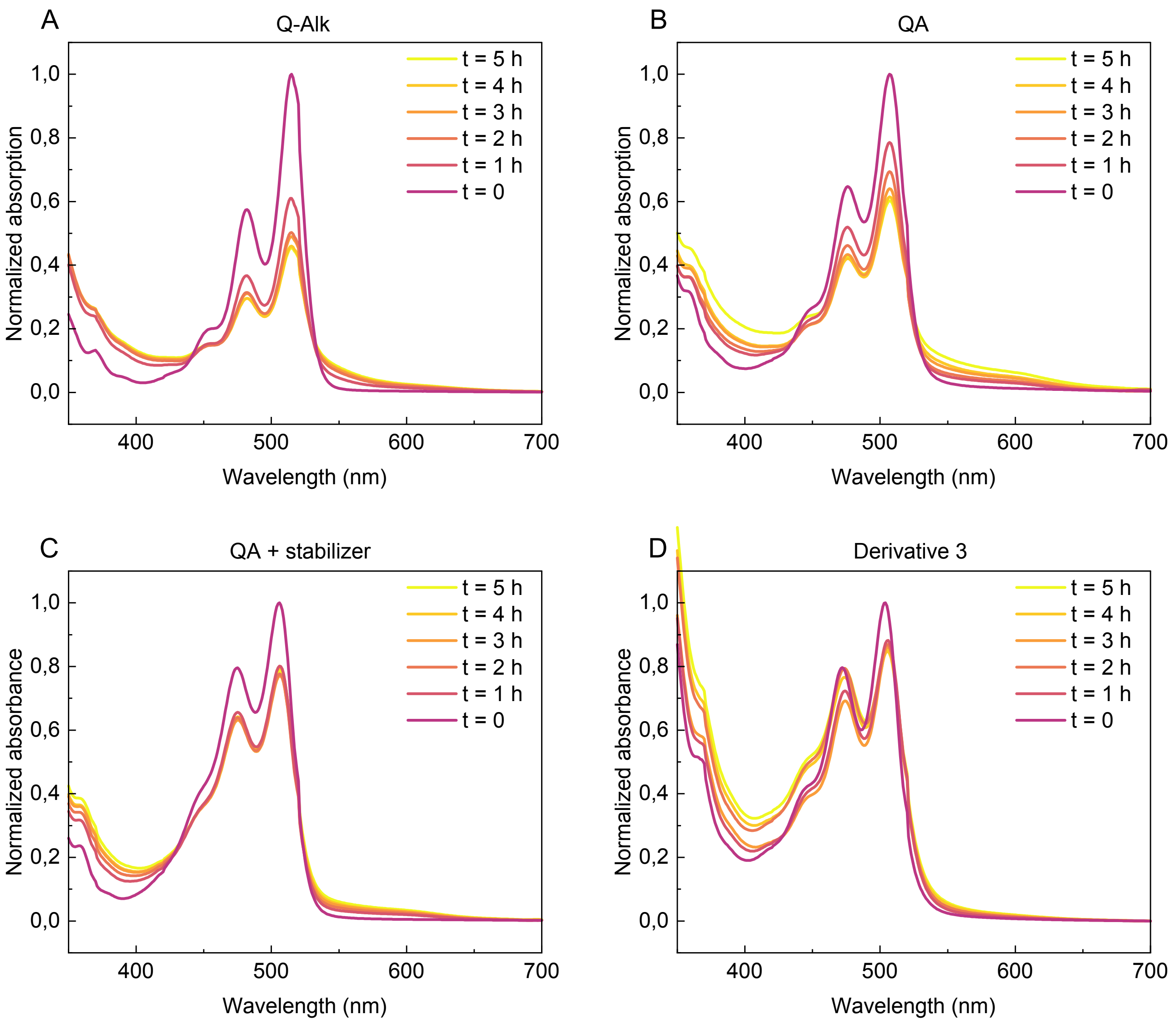

Figure S32. Absorption spectra as a function of irradiation exposure time of a PMMA slab containing A) **Q-Alk** (quinacridone alkylated with ethylhexyl chains); B) quinacridone (**QA**); C) quinacridone in the presence of bis(1-octyloxy-2,2,6,6-tetramethyl-4-piperidyl) sebacate as stabilizer; D) derivative **3**. Values of absorbance at maximum absorption wavelength (λ_{max}) were used to obtain the graph in Figure 5 of the main text.

7. DFT studies

All the calculations were carried out using the Gaussian 16 program package, revision C.01 installed on the HPC infrastructure Galileo100 at CINECA facility (Italy).[S7] In our investigation, the level of theory chosen for the optimization of the reported stationary points was DFT (Density Functional Theory) by using the B3LYP functional and the 6-311+G(2d,2p) basis set in the gas phase. No symmetry constraint was applied to the structures investigated and a thorough conformers search has been performed to locate the absolute minimum for each species. The structures and data used for this work correspond to those of the absolute minimum. Frequency calculations were performed in the gas phase to check that the located stationary states had 0 imaginary frequencies, thus confirming their nature as minima along the potential energy surface. Hexyl groups were replaced with methyl groups in molecules **10** and **12**. *Tert*-butyl groups were replaced with methyl groups in molecules **9** and **11** for simplicity.

Table S1. Experimental and computed energy levels (in eV) for derivatives **9-12**.

Entry	E_{HOMO}[a]	E_{LUMO}[a]	E_g[a]	E_{HOMO}[b]	E_{LUMO}[b]	E_g[b]
**9**	-5.80	-3.17	-2.63	-6.14	-2.63	3.51
**10**	-5.46	-3.06	-2.40	-5.42	-2.44	2.98
**11**	-5.80	-3.20	-2.60	-6.43	-2.89	3.54
**12**	-5.69	-3.03	-2.66	-5.60	-2.60	3.00

[a] From cyclic voltammetry; [b] from DFT calculations.

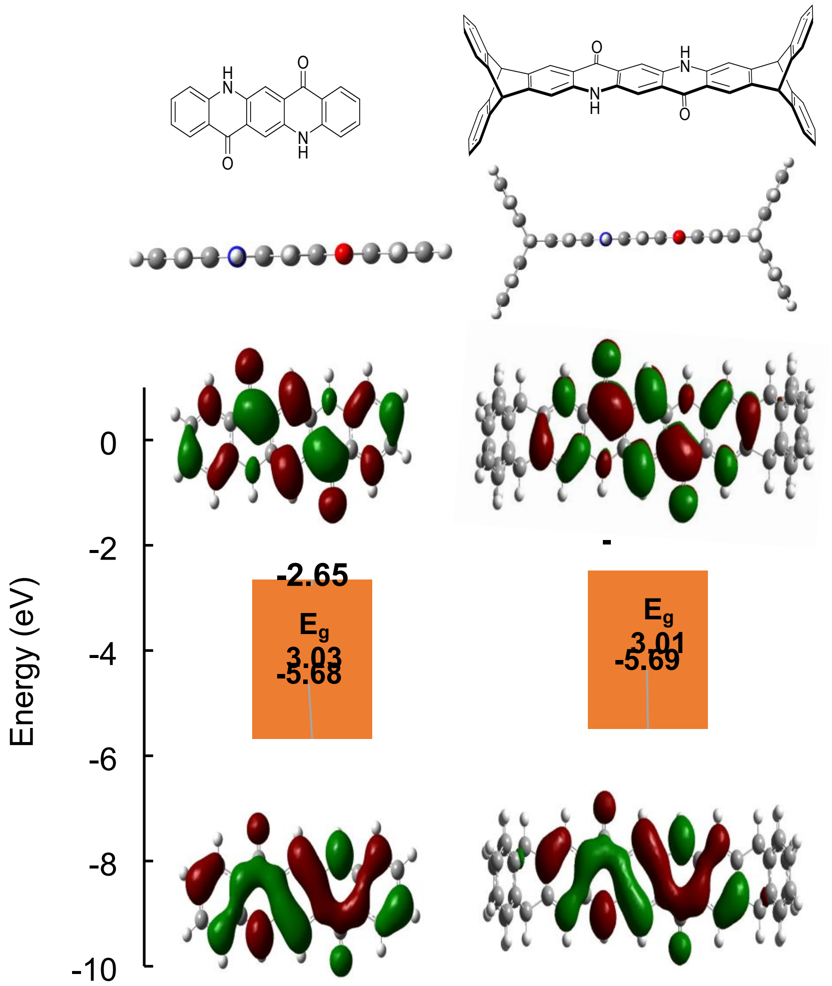

Figure S33. Optimized molecular structure and calculated molecular orbitals of classic quinacridone (**QA**) and triptycene-fused quinacridone **3**.

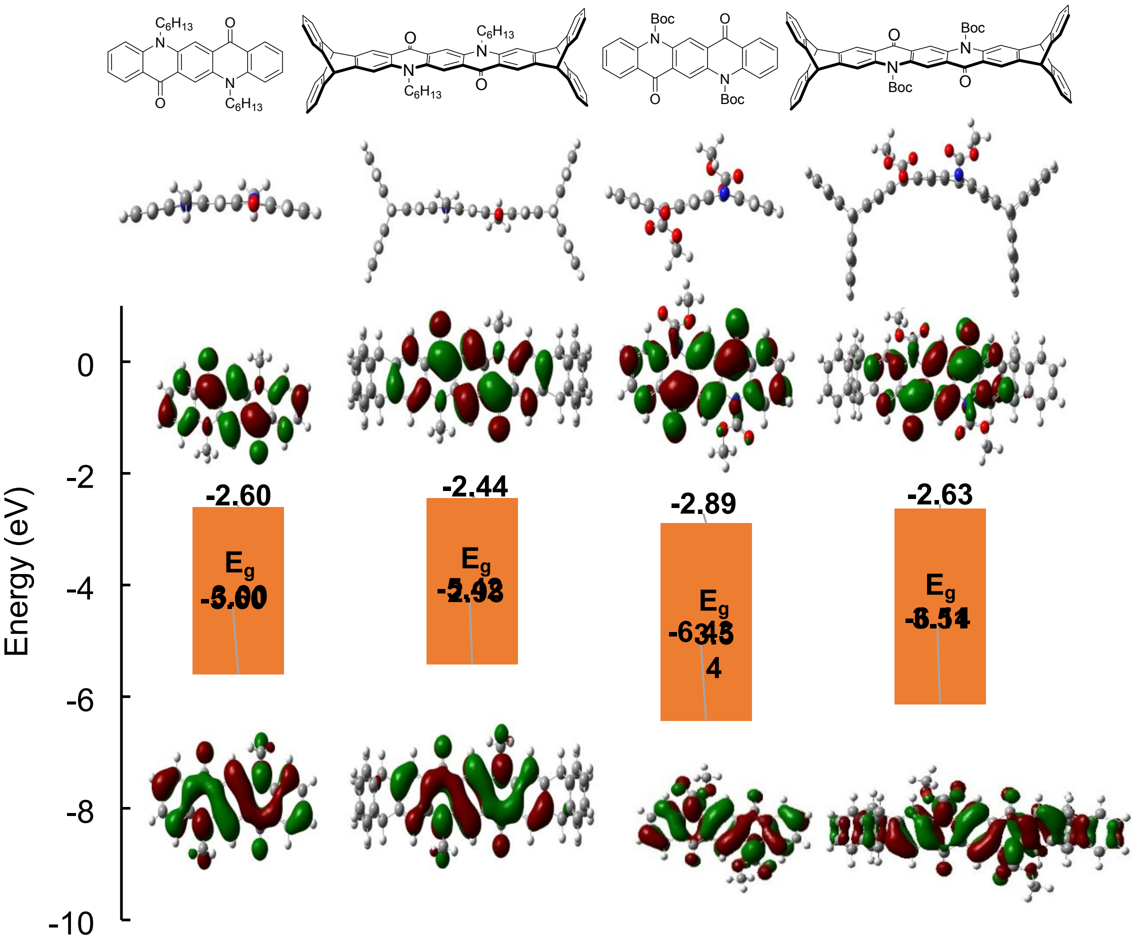

Figure S34. Optimized molecular structures and calculated molecular orbitals of classic Quinacridone derivatives and Triptycene-fused Quinacridone derivatives.

Optimized geometry listed in cartesian format (coordinates are given in Å) are reported below.

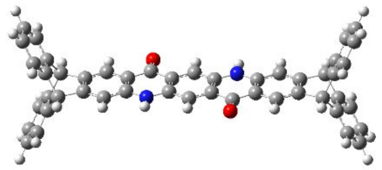

Compound 3

C	7.53684	0.00009	1.26309
C	8.22179	-1.22222	0.64844
C	8.18160	-1.22379	-0.75673
C	7.46761	0.00007	-1.33560
C	8.22160	1.22250	0.64843
C	8.18141	1.22405	-0.75674
C	6.07373	-0.00003	-0.70796
C	6.11088	-0.00002	0.70962
C	4.93565	-0.00011	1.42543
C	3.69107	-0.00022	0.75773
C	3.66421	-0.00021	-0.65531
C	4.87409	-0.00013	-1.38711
C	8.75407	-2.26595	-1.47561
C	9.37480	-3.31540	-0.78580
C	9.41495	-3.31450	0.60787
C	8.83591	-2.26442	1.33219
C	8.83556	2.26480	1.33217
C	9.41443	3.31497	0.60784
C	9.37429	3.31585	-0.78582
C	8.75372	2.26630	-1.47563
C	2.44391	-0.00035	1.53755
C	1.19190	-0.00032	0.74246
C	1.23202	-0.00031	-0.67602
N	2.45704	-0.00027	-1.31967
C	-0.03794	-0.00032	1.39862
C	-1.23202	-0.00032	0.67601
C	-1.19190	-0.00033	-0.74246
C	0.03794	-0.00032	-1.39862
N	-2.45704	-0.00029	1.31967
C	-2.44391	-0.00038	-1.53755
O	2.42685	-0.00013	2.77158
O	-2.42685	-0.00012	-2.77158
C	-7.46761	0.00007	1.33560
C	-6.07373	-0.00004	0.70796
C	-6.11088	-0.00003	-0.70962
C	-7.53684	0.00009	-1.26309
C	-8.18160	-1.22379	0.75673
C	-8.22180	-1.22221	-0.64844
C	-8.22160	1.22250	-0.64843
C	-8.18140	1.22406	0.75674
C	-8.75371	2.26630	1.47563

C	-9.37427	3.31586	0.78583
C	-9.41442	3.31498	-0.60784
C	-8.83555	2.26481	-1.33217
C	-4.93565	-0.00012	-1.42543
C	-3.69107	-0.00024	-0.75773
C	-3.66421	-0.00023	0.65531
C	-4.87409	-0.00014	1.38711
C	-8.75408	-2.26594	1.47561
C	-9.37482	-3.31539	0.78580
C	-9.41496	-3.31449	-0.60787
C	-8.83593	-2.26441	-1.33219
H	7.56470	0.00010	2.35466
H	7.44173	0.00007	-2.42762
H	4.91783	-0.00012	2.51074
H	4.84827	-0.00013	-2.47434
H	8.72236	-2.26663	-2.56203
H	9.82598	-4.13244	-1.34093
H	9.89684	-4.13153	1.13662
H	8.86611	-2.26529	2.41850
H	8.86576	2.26568	2.41848
H	9.89620	4.13208	1.13659
H	9.82534	4.13296	-1.34096
H	8.72201	2.26696	-2.56205
H	-0.02465	-0.00032	2.48465
H	0.02465	-0.00032	-2.48466
H	-7.44173	0.00007	2.42762
H	-7.56470	0.00010	-2.35466
H	-8.72200	2.26697	2.56205
H	-9.82532	4.13297	1.34096
H	-9.89618	4.13209	-1.13658
H	-8.86575	2.26569	-2.41848
H	-4.91783	-0.00013	-2.51074
H	-4.84827	-0.00014	2.47434
H	-8.72237	-2.26662	2.56203
H	-9.82600	-4.13243	1.34093
H	-9.89686	-4.13152	-1.13662
H	-8.86612	-2.26528	-2.41850
H	2.46252	-0.00026	-2.32883
H	-2.46252	-0.00028	2.32883

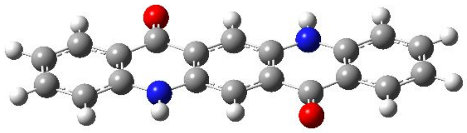

Quinacridone

C	-6.14946	0.39425	0.00002
C	-6.03956	-1.00809	0.00002
C	-4.79952	-1.62433	0.00001
C	-3.62728	-0.84209	0.00000
C	-3.72646	0.56911	0.00001
C	-5.00035	1.16536	0.00001
N	-2.38749	-1.44355	-0.00000
C	-1.19706	-0.73784	-0.00001
C	-1.22733	0.68086	-0.00001
C	-2.51773	1.41266	-0.00000
C	0.03242	-1.39862	-0.00002
C	1.22733	-0.68086	-0.00003
C	1.19706	0.73784	-0.00002
C	-0.03242	1.39863	-0.00001
C	2.51773	-1.41266	-0.00005
C	3.72646	-0.56911	-0.00001
C	3.62728	0.84209	-0.00001
N	2.38749	1.44356	-0.00002
C	5.00035	-1.16536	0.00001
C	6.14946	-0.39425	0.00003
C	6.03956	1.00809	0.00003
C	4.79952	1.62433	0.00001
O	2.56342	-2.64447	0.00002
O	-2.56342	2.64447	-0.00000
H	-7.12791	0.86328	0.00002
H	-6.93623	-1.62101	0.00002
H	-4.72130	-2.70852	0.00001
H	-5.03842	2.24966	0.00001
H	-2.34127	-2.45179	-0.00001
H	0.10077	-2.48264	-0.00002
H	-0.10077	2.48265	-0.00001
H	2.34127	2.45179	-0.00001
H	5.03842	-2.24966	0.00001
H	7.12791	-0.86329	0.00005
H	6.93623	1.62100	0.00005
H	4.72130	2.70852	0.00001

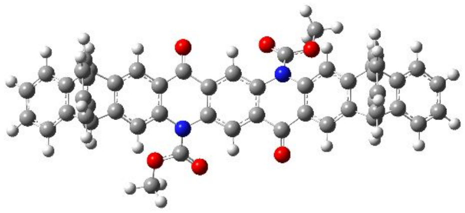

Compound 9

C	-7.10019	0.94343	1.02846
C	-8.25085	0.15599	0.39637
C	-8.21879	0.17099	-1.00883
C	-7.03997	0.96908	-1.57004
C	-7.18043	2.35550	0.44318
C	-7.15009	2.36951	-0.96195
C	-5.78650	0.34937	-0.95190
C	-5.82165	0.32796	0.45894
C	-4.77382	-0.19568	1.19402
C	-3.65326	-0.72612	0.52580
C	-3.59265	-0.65817	-0.87821
C	-4.67488	-0.12959	-1.61006
C	-9.19416	-0.49667	-1.73923
C	-10.21005	-1.18298	-1.06137
C	-10.24177	-1.19736	0.33242
C	-9.25726	-0.52608	1.06905
C	-7.27526	3.54442	1.15586
C	-7.34213	4.75814	0.45942
C	-7.31189	4.77233	-0.93436
C	-7.21467	3.57341	-1.65259
N	-2.55374	-1.28358	1.24806
C	-1.25951	-1.23802	0.64282
C	-1.15991	-1.19675	-0.76452
C	-2.37208	-1.05417	-1.60985
C	-0.08677	-1.21095	1.39480
C	1.15992	-1.19675	0.76456
C	1.25952	-1.23805	-0.64277
C	0.08678	-1.21098	-1.39476
C	2.37210	-1.05415	1.60987
N	2.55375	-1.28362	-1.24803
C	7.03999	0.96910	1.57001
C	5.78651	0.34938	0.95188
C	5.82165	0.32796	-0.45896
C	7.10018	0.94343	-1.02849
C	7.15009	2.36953	0.96190
C	7.18042	2.35551	-0.44323
C	8.25086	0.15601	-0.39641
C	8.21881	0.17101	1.00880
C	9.19419	-0.49664	1.73920
C	10.21007	-1.18295	1.06132
C	10.24178	-1.19734	-0.33246
C	9.25726	-0.52607	-1.06908
C	4.77382	-0.19569	-1.19403
C	3.65327	-0.72614	-0.52579

C	3.59267	-0.65816	0.87821
C	4.67490	-0.12958	1.61005
C	7.21467	3.57344	1.65253
C	7.31188	4.77235	0.93429
C	7.34211	4.75815	-0.45949
C	7.27524	3.54442	-1.15592
O	-2.33758	-1.15729	-2.83285
O	2.33762	-1.15730	2.83287
C	-2.72504	-2.14080	2.35227
C	2.72502	-2.14093	-2.35218
O	1.81779	-2.62804	-2.99189
O	4.02576	-2.38644	-2.60678
C	4.25724	-3.27659	-3.71313
O	-1.81783	-2.62772	2.99216
O	-4.02578	-2.38641	2.60675
C	-4.25730	-3.27641	3.71322
H	-7.12670	0.93288	2.12034
H	-7.01217	0.97911	-2.66161
H	-4.81431	-0.19879	2.27481
H	-4.58285	-0.10496	-2.69122
H	-9.16900	-0.48706	-2.82566
H	-10.97574	-1.70631	-1.62638
H	-11.03262	-1.73099	0.85132
H	-9.28191	-0.53713	2.15563
H	-7.29798	3.53343	2.24246
H	-7.41728	5.69134	1.00977
H	-7.36322	5.71677	-1.46802
H	-7.18959	3.58508	-2.73901
H	-0.09948	-1.19733	2.47423
H	0.09949	-1.19739	-2.47419
H	7.01219	0.97915	2.66157
H	7.12668	0.93287	-2.12038
H	9.16904	-0.48702	2.82562
H	10.97577	-1.70627	1.62633
H	11.03263	-1.73096	-0.85136
H	9.28190	-0.53713	-2.15567
H	4.81430	-0.19881	-2.27482
H	4.58288	-0.10493	2.69121
H	7.18960	3.58511	2.73895
H	7.36321	5.71680	1.46795
H	7.41725	5.69134	-1.00984
H	7.29795	3.53342	-2.24251
H	5.34101	-3.35232	-3.79574
H	3.81720	-4.25589	-3.51422
H	3.82496	-2.87223	-4.63087
H	-5.34107	-3.35218	3.79576
H	-3.81720	-4.25572	3.51448
H	-3.82510	-2.87191	4.63093

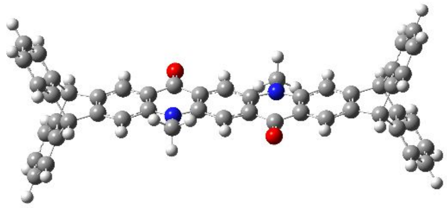

Compound 10

C	-7.51243	-0.12974	-1.31517
C	-8.14645	-1.33098	-0.61032
C	-8.12879	-1.21564	0.79064
C	-7.48558	0.08591	1.27516
C	-8.26763	1.10204	-0.81192
C	-8.25080	1.22019	0.58891
C	-6.08360	0.10909	0.66515
C	-6.09928	-0.01343	-0.74282
C	-4.90710	-0.03654	-1.42816
C	-3.67964	0.07547	-0.74104
C	-3.66198	0.22649	0.66841
C	-4.89880	0.22403	1.36414
C	-8.66077	-2.22325	1.58577
C	-9.21753	-3.35572	0.97735
C	-9.23526	-3.47065	-0.41197
C	-8.69733	-2.45515	-1.21328
C	-8.92199	2.05206	-1.58660
C	-9.56483	3.12668	-0.95845
C	-9.54781	3.24331	0.43077
C	-8.88672	2.28684	1.21223
C	-2.43640	0.01756	-1.51709
C	-1.19556	0.02032	-0.71420
C	-1.23607	0.18166	0.69523
N	-2.45631	0.37081	1.35035
C	0.01758	-0.14628	-1.38180
C	1.23607	-0.18163	-0.69522
C	1.19557	-0.02031	0.71421
C	-0.01757	0.14630	1.38180
N	2.45629	-0.37079	-1.35035
C	2.43641	-0.01757	1.51709
O	-2.41766	-0.06277	-2.74924
O	2.41768	0.06274	2.74924
C	7.48558	-0.08588	-1.27516
C	6.08360	-0.10909	-0.66515
C	6.09929	0.01340	0.74282
C	7.51243	0.12970	1.31518
C	8.25081	-1.22018	-0.58895
C	8.26764	-1.10207	0.81189
C	8.14646	1.33097	0.61036
C	8.12879	1.21566	-0.79060
C	8.66077	2.22329	-1.58571
C	9.21752	3.35575	-0.97726
C	9.23525	3.47064	0.41206
C	8.69732	2.45512	1.21334

C	4.90710	0.03650	1.42816
C	3.67965	-0.07550	0.74103
C	3.66198	-0.22650	-0.66843
C	4.89881	-0.22401	-1.36415
C	8.88674	-2.28680	-1.21229
C	9.54783	-3.24329	-0.43086
C	9.56485	-3.12670	0.95836
C	8.92200	-2.05210	1.58654
C	-2.45995	0.72646	2.76720
C	2.45984	-0.72643	-2.76721
H	2.50778	0.15410	-3.41934
H	-2.50788	-0.15407	3.41933
H	-7.52135	-0.22055	-2.40338
H	-7.47770	0.17654	2.36386
H	-4.85837	-0.14352	-2.50723
H	-4.92944	0.28167	2.44490
H	-8.64653	-2.13363	2.66893
H	-9.63647	-4.14664	1.59250
H	-9.66758	-4.35153	-0.87743
H	-8.70999	-2.54613	-2.29619
H	-8.93417	1.96271	-2.66965
H	-10.07831	3.87209	-1.55862
H	-10.04865	4.07869	0.91117
H	-8.87330	2.37761	2.29532
H	-0.05640	-0.22631	-2.45851
H	0.05641	0.22630	2.45852
H	7.47771	-0.17648	-2.36387
H	7.52136	0.22048	2.40339
H	8.64652	2.13370	-2.66887
H	9.63646	4.14669	-1.59239
H	9.66757	4.35151	0.87755
H	8.70998	2.54607	2.29626
H	4.85837	0.14347	2.50723
H	4.92946	-0.28160	-2.44491
H	8.87331	-2.37755	-2.29538
H	10.04868	-4.07866	-0.91128
H	10.07833	-3.87213	1.55852
H	8.93418	-1.96278	2.66960
H	-1.55872	1.28980	3.00365
H	-3.31022	1.37420	2.97937
H	1.55856	-1.28972	-3.00360
H	3.31007	-1.37422	-2.97944

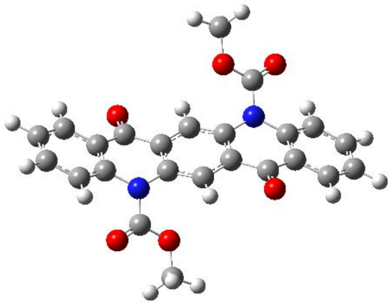

Compound 11

C	5.94227	0.87795	0.79273
C	5.69185	2.13653	0.23427
C	4.44443	2.39344	-0.31041
C	3.44770	1.40529	-0.31907
C	3.71751	0.12577	0.21323
C	4.97433	-0.12120	0.78831
C	2.10900	1.75746	-0.83408
C	1.03125	0.81620	-0.44261
C	1.34046	-0.45873	0.07608
N	2.70208	-0.87997	0.14698
C	-0.29134	1.25295	-0.53363
C	-1.34045	0.45874	-0.07608
C	-1.03124	-0.81619	0.44260
C	0.29134	-1.25294	0.53363
N	-2.70208	0.87998	-0.14699
C	-2.10900	-1.75745	0.83407
O	1.88147	2.79506	-1.45024
O	-1.88146	-2.79506	1.45022
C	-5.94225	-0.87795	-0.79278
C	-5.69183	-2.13653	-0.23432
C	-4.44441	-2.39344	0.31036
C	-3.44768	-1.40529	0.31905
C	-3.71750	-0.12577	-0.21325
C	-4.97431	0.12121	-0.78834
C	3.08823	-2.20779	-0.13552
O	2.06631	-2.93148	-0.62591
O	4.20804	-2.64929	0.01261
C	2.38411	-4.30555	-0.91490
C	-3.08824	2.20778	0.13554
O	-2.06633	2.93149	0.62593
C	-2.38417	4.30553	0.91499
O	-4.20807	2.64926	-0.01253
H	6.90549	0.66872	1.24860
H	6.45894	2.90426	0.24210
H	4.19049	3.35821	-0.73632
H	5.18961	-1.08720	1.21673
H	-0.45356	2.23442	-0.95551
H	0.45357	-2.23441	0.95550
H	-6.90547	-0.66872	-1.24866
H	-6.45891	-2.90427	-0.24217
H	-4.19048	-3.35822	0.73627
H	-5.18960	1.08721	-1.21676

H	-3.14399	4.36385	1.69701
H	-1.44796	4.74846	1.25205
H	-2.75136	4.81150	0.01985
H	1.44790	-4.74846	-1.25195
H	3.14394	-4.36392	-1.69691
H	2.75128	-4.81148	-0.01973

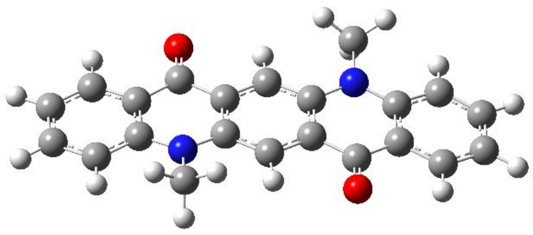

Compound 12

H	-2.50065	-3.36815	-0.53824
H	2.50066	3.36815	-0.53824
H	-1.65891	-3.08998	1.00259
H	-3.41256	-3.05713	0.95955
H	1.65892	3.08999	1.00258
H	3.41257	3.05712	0.95955
H	-4.82414	2.54507	-0.22880
H	-7.01524	1.33835	-0.46465
H	-7.01270	-1.15673	-0.45288
H	-4.94370	-2.40654	-0.20051
H	-0.02662	2.46887	0.06637
H	0.02662	-2.46887	0.06637
H	4.94370	2.40654	-0.20051
H	4.82414	-2.54507	-0.22880
H	7.01523	-1.33835	-0.46465
H	7.01270	1.15673	-0.45288
C	-2.51563	-2.79684	0.39781
C	2.51564	2.79684	0.39781
O	-2.39683	2.76734	0.07042
O	2.39683	-2.76734	0.07042
C	-4.88284	1.46189	-0.21221
C	-6.08581	0.79192	-0.34262
C	-6.08185	-0.61063	-0.32903
C	-4.90396	-1.32554	-0.17202
C	-2.42453	1.53415	0.05381
C	-3.67229	0.76544	-0.05541
C	-3.67001	-0.65285	-0.01493
N	-2.48135	-1.35411	0.17056
C	0.03416	1.38924	0.11113
C	-1.19064	0.72220	0.10986
C	-1.24902	-0.69559	0.13683
C	-0.03416	-1.38924	0.11113
N	2.48135	1.35411	0.17056
C	1.24902	0.69559	0.13683
C	1.19064	-0.72220	0.10986
C	2.42452	-1.53415	0.05381
C	4.90396	1.32554	-0.17202
C	3.67001	0.65285	-0.01493
C	3.67229	-0.76544	-0.05541
C	4.88284	-1.46189	-0.21221
C	6.08581	-0.79192	-0.34262
C	6.08184	0.61063	-0.32903

8. Copies of NMR and mass spectra of new compounds

Compound 7

^{1}H-NMR (300 MHz, DMSO)

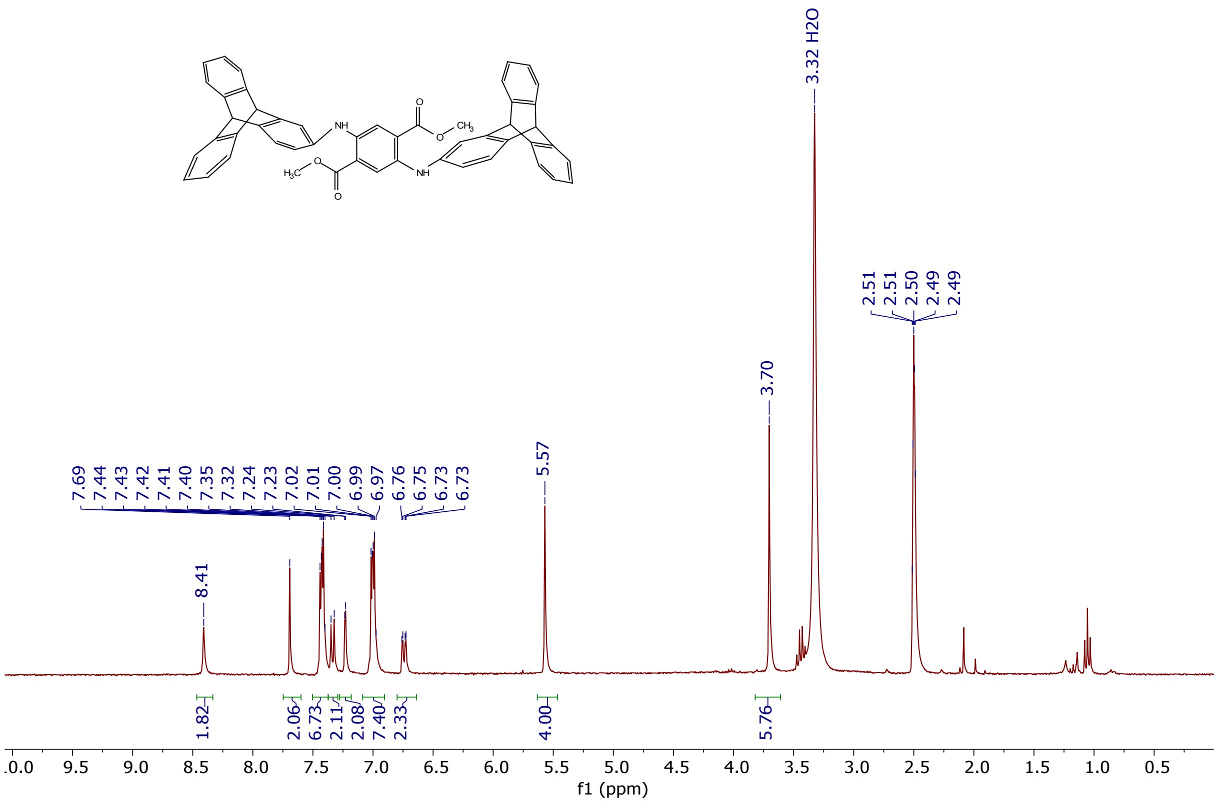

^{13}C-NMR (75 MHz, DMSO)

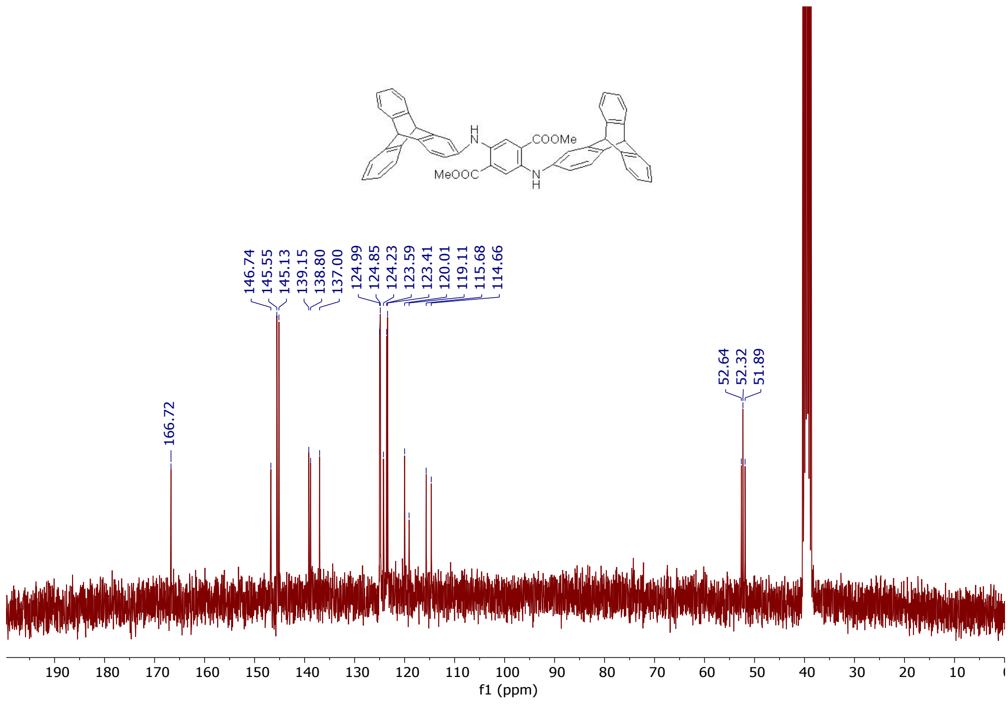

ESI-MS

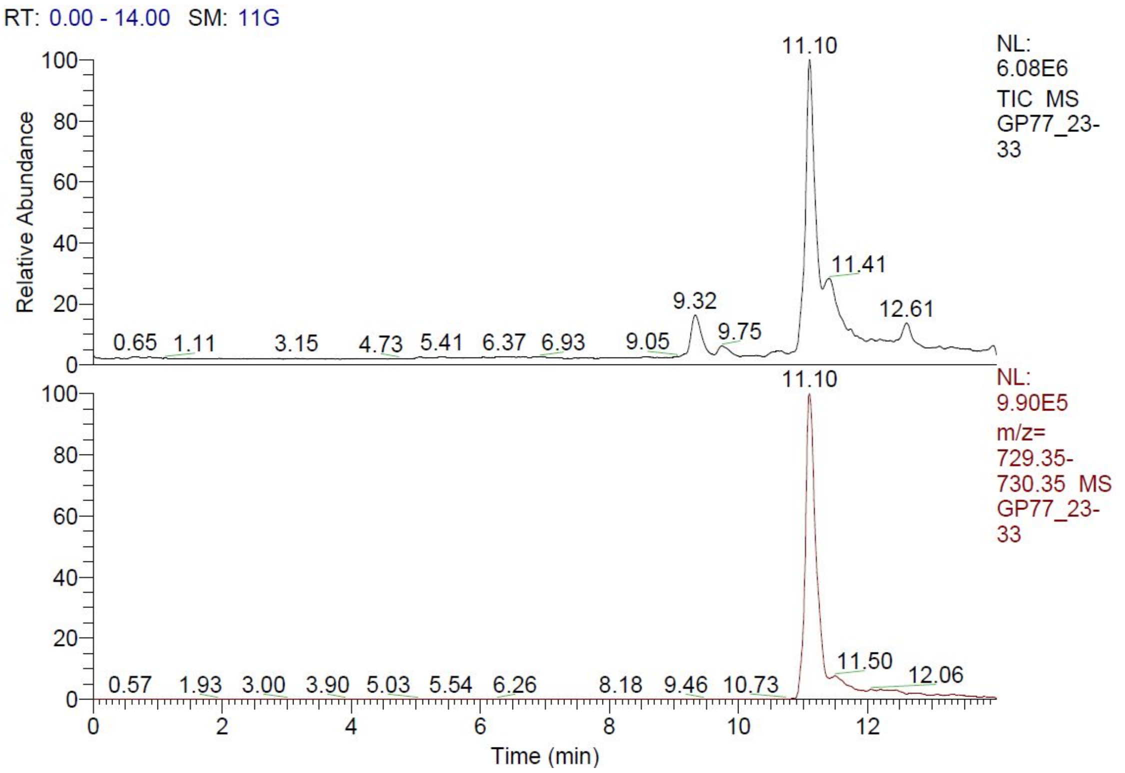
RT: 0.00 - 14.00 SM: 11G
NL: 6.08E6
TIC MS GP77_23-33
NL: 9.90E5
m/z= 729.35-730.35 MS GP77_23-33
Relative Abundance
Time (min)
11.10
11.41
9.32
12.61
9.75
0.65
1.11
3.15
4.73
5.41
6.37
6.93
9.05
11.50
12.06
0.57
1.93
3.00
3.90
5.03
5.54
6.26
8.18
9.46
10.73

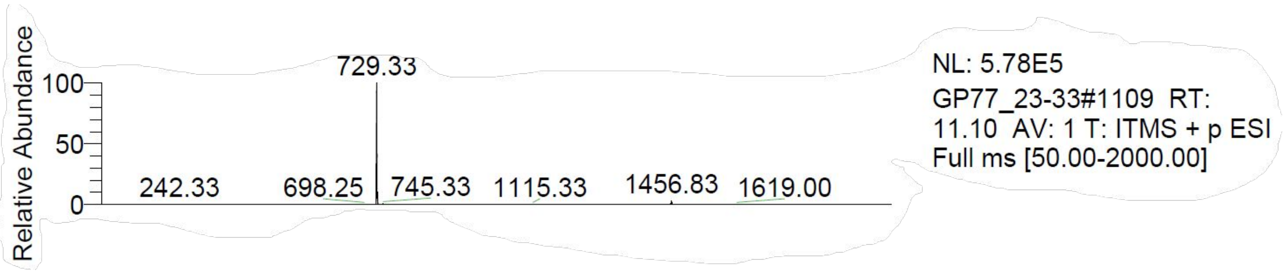
NL: 5.78E5
GP77_23-33#1109 RT: 11.10 AV: 1 T: ITMS + p ESI Full ms [50.00-2000.00]
Relative Abundance
729.33
242.33
698.25
745.33
1115.33
1456.83
1619.00

Compound 8

^{1}H-NMR (300 MHz, DMSO)

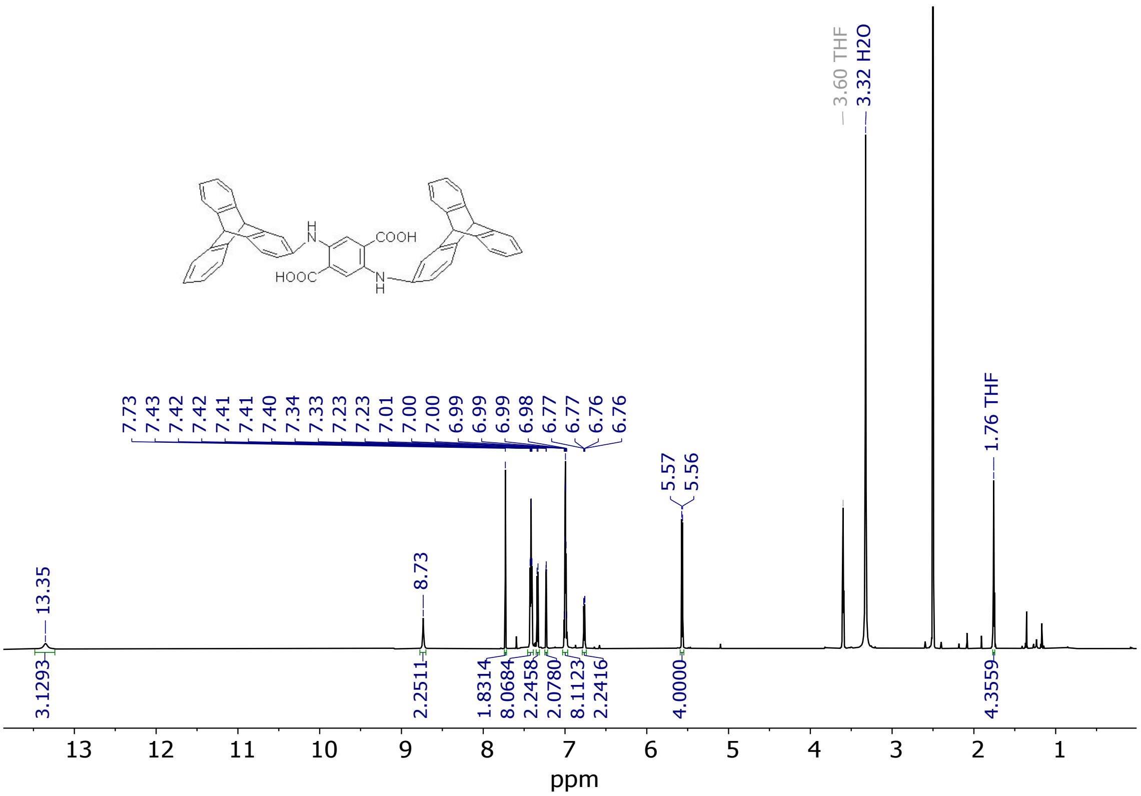

^{13}C-NMR (75 MHz, DMSO)

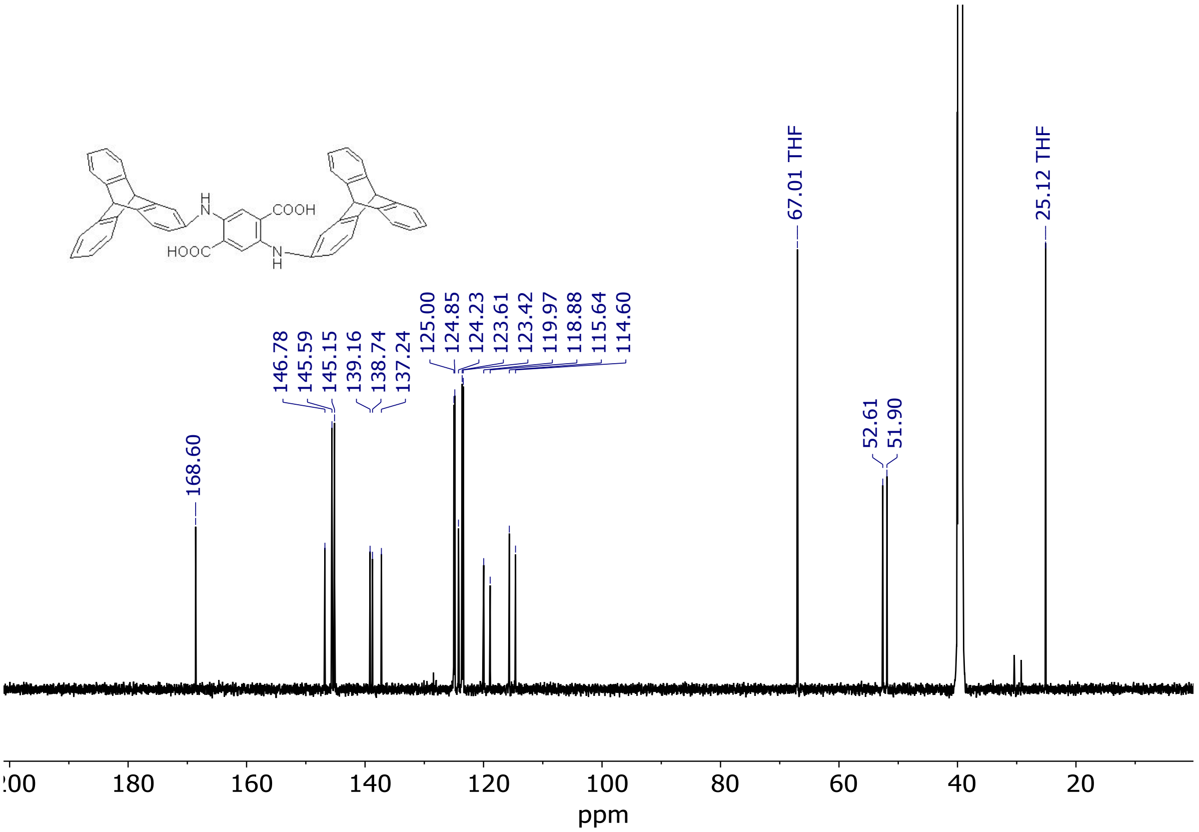

ESI-MS

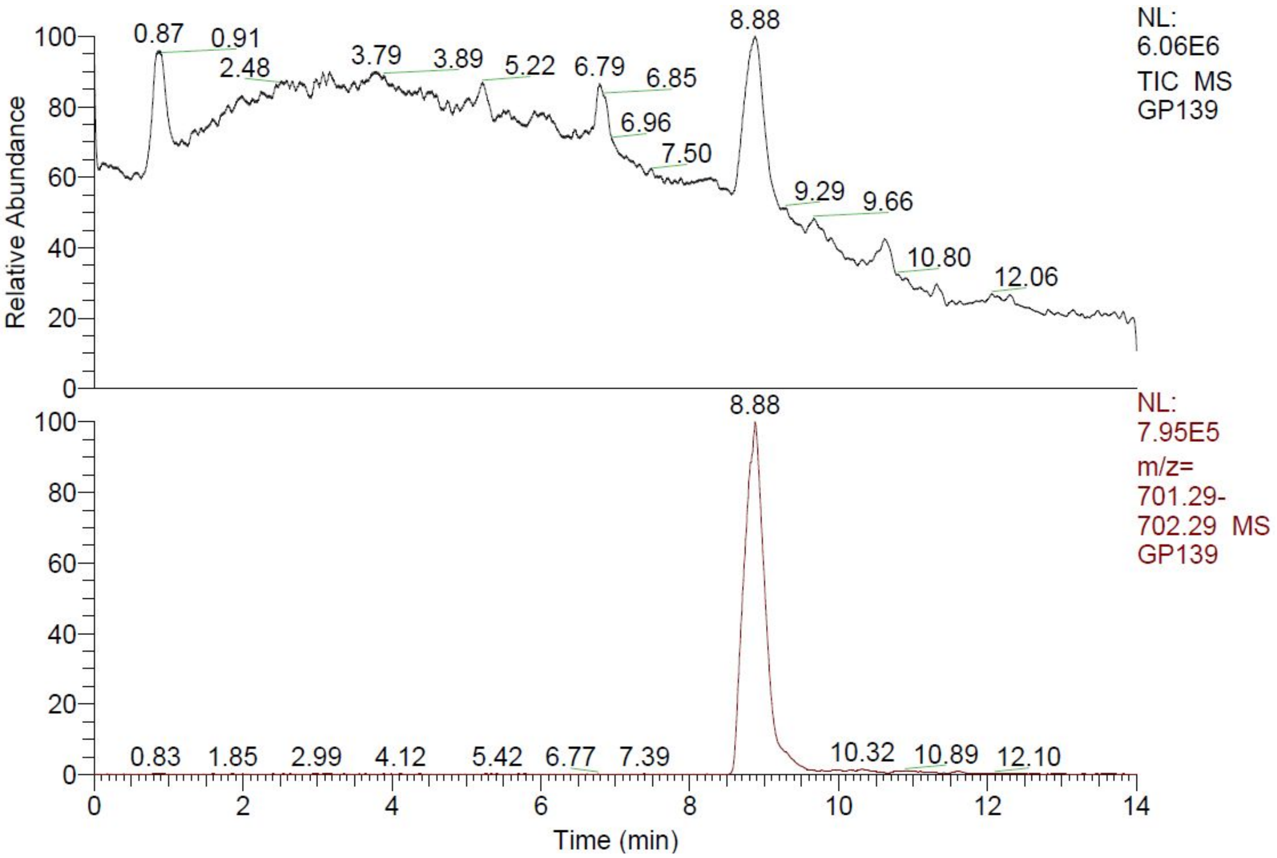

NL:
6.06E6
TIC MS
GP139
NL:
7.95E5
m/z=
701.29-
702.29 MS
GP139
Relative Abundance
Time (min)
0.87
0.91
2.48
3.79
3.89
5.22
6.79
6.85
6.96
7.50
8.88
9.29
9.66
10.80
12.06
0.83
1.85
2.99
4.12
5.42
6.77
7.39
8.88
10.32
10.89
12.10

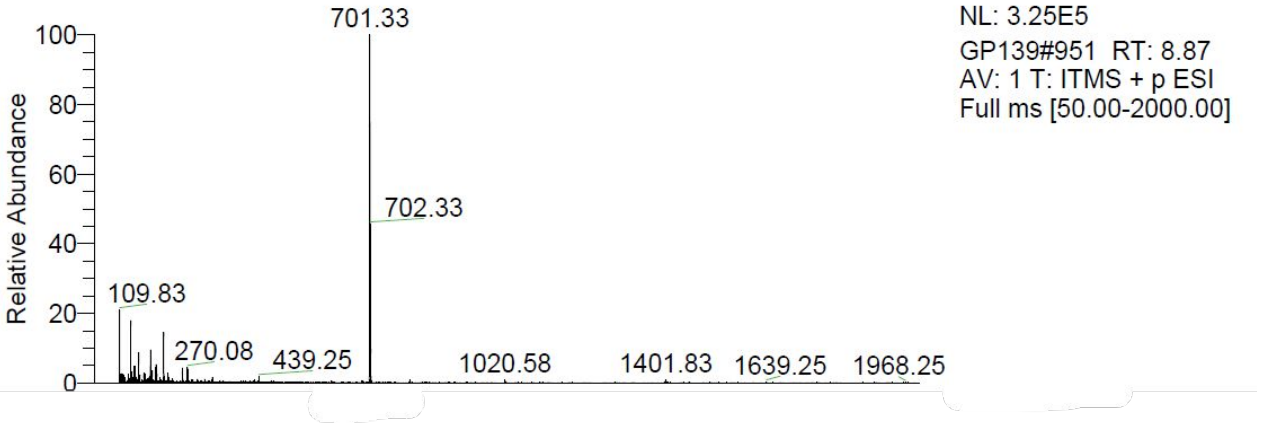

NL: 3.25E5
GP139#951 RT: 8.87
AV: 1 T: ITMS + p ESI
Full ms [50.00-2000.00]
Relative Abundance
701.33
702.33
109.83
270.08
439.25
1020.58
1401.83
1639.25
1968.25

Compound 3

^{1}H-NMR (400 MHz, DMSO)

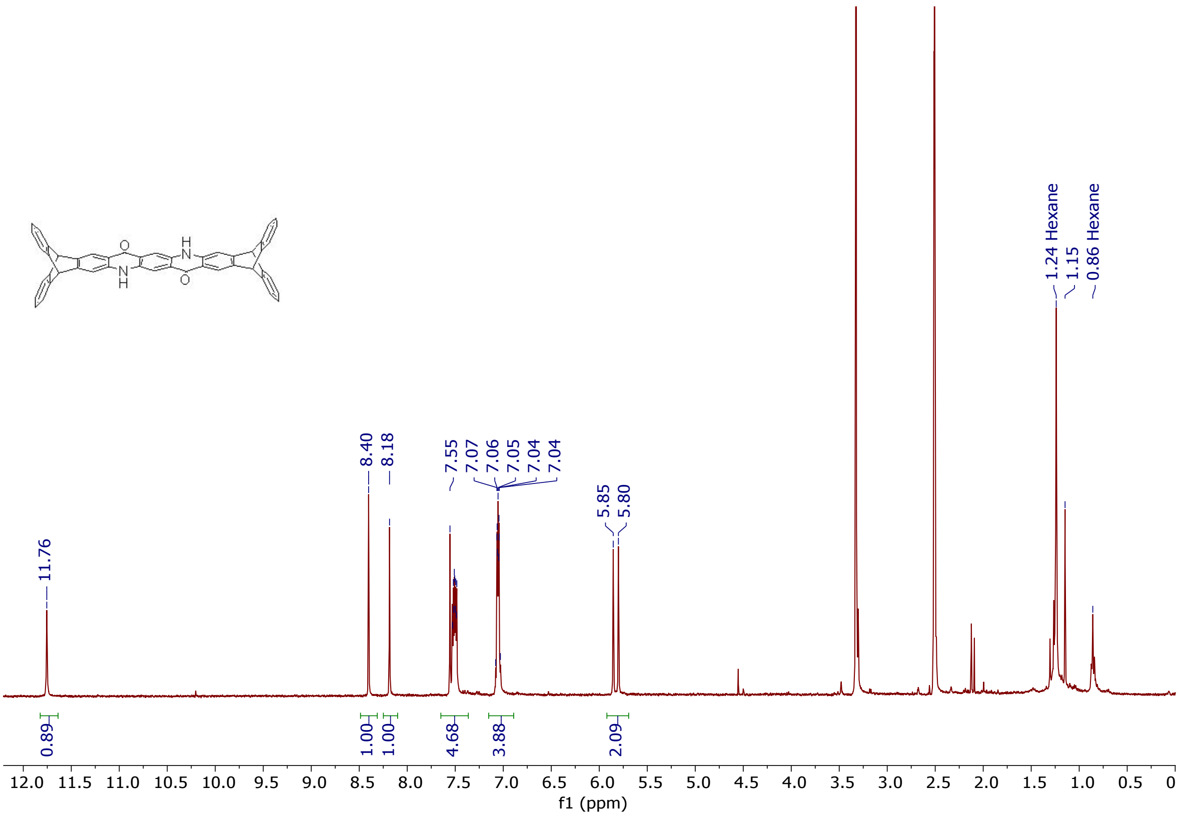

^{13}C-NMR (75 MHz, DMSO)

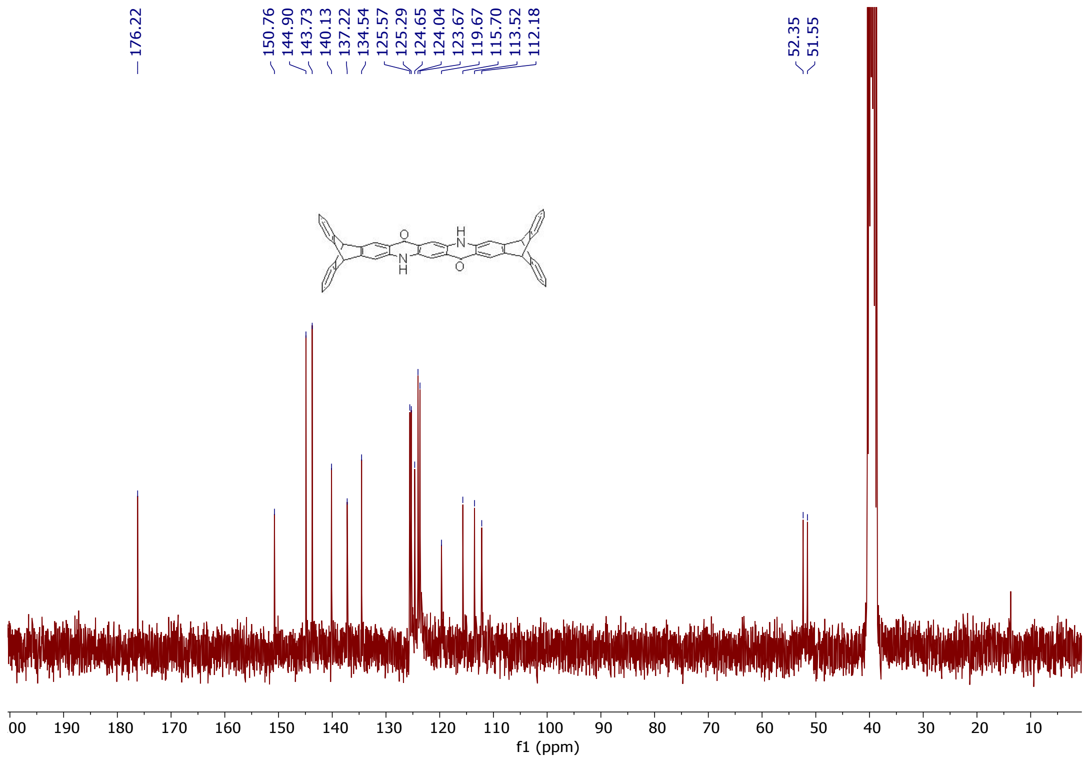

COSY

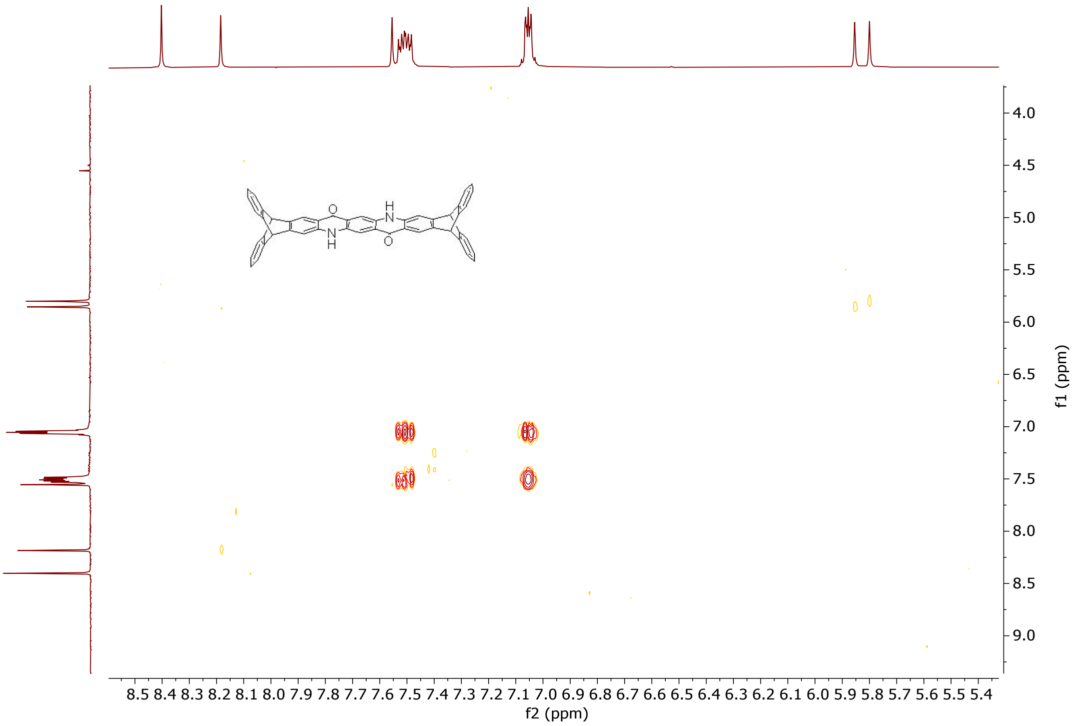

HSQC

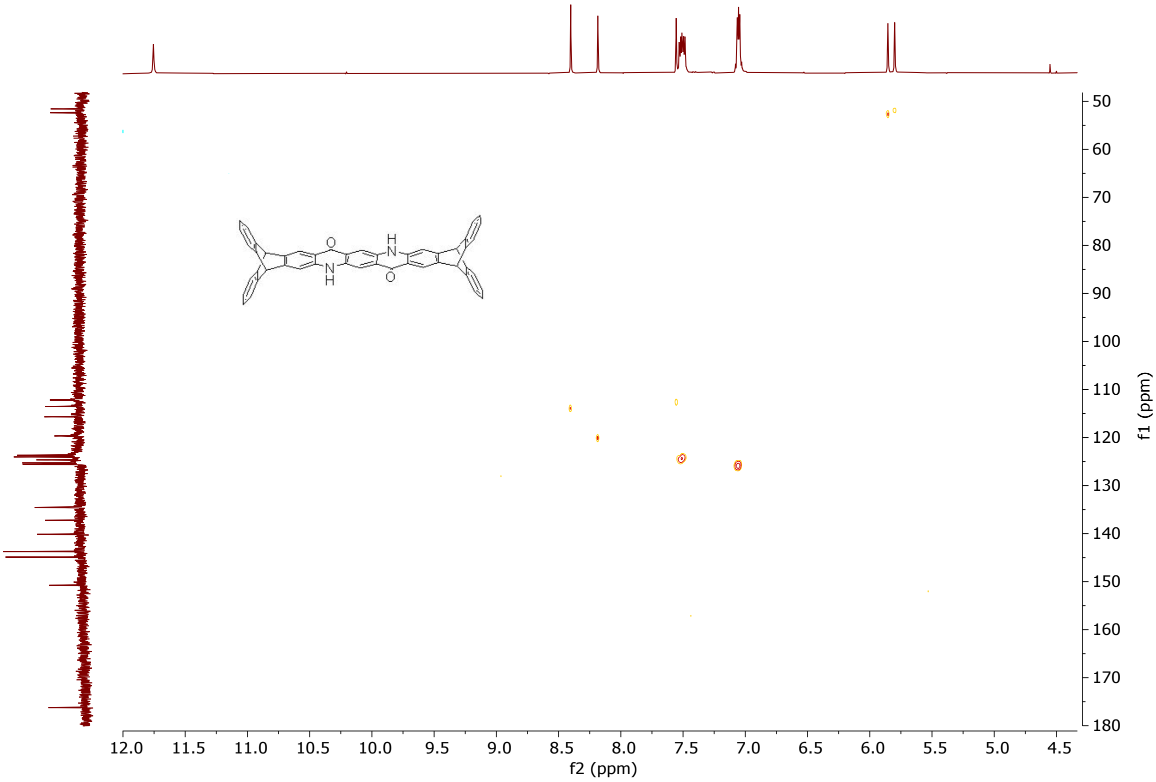

ESI-MS

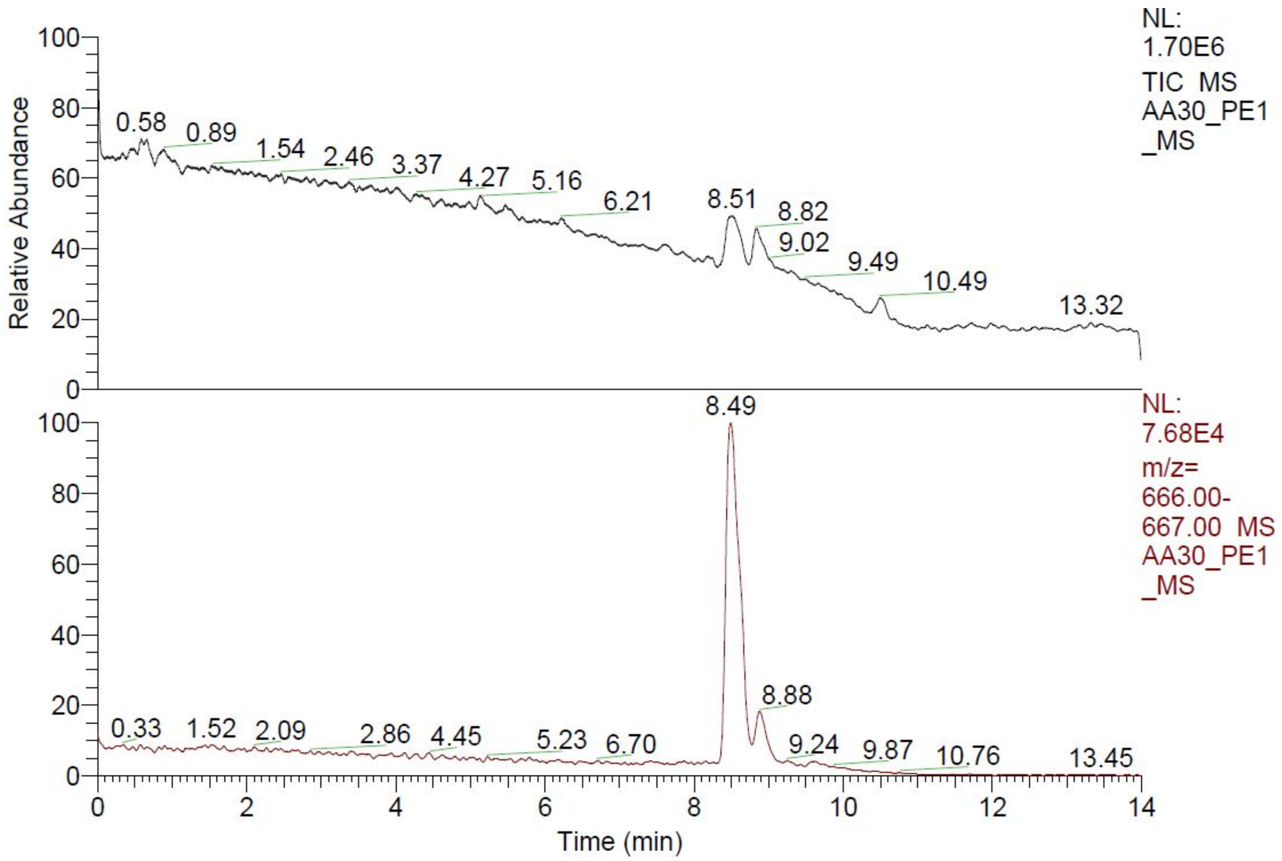

NL: 1.70E6
TIC MS AA30_PE1_MS
NL: 7.68E4
m/z= 666.00-667.00 MS AA30_PE1_MS
Relative Abundance
Time (min)
0.58
0.89
1.54
2.46
3.37
4.27
5.16
6.21
8.51
8.82
9.02
9.49
10.49
13.32
8.49
0.33
1.52
2.09
2.86
4.45
5.23
6.70
8.88
9.24
9.87
10.76
13.45

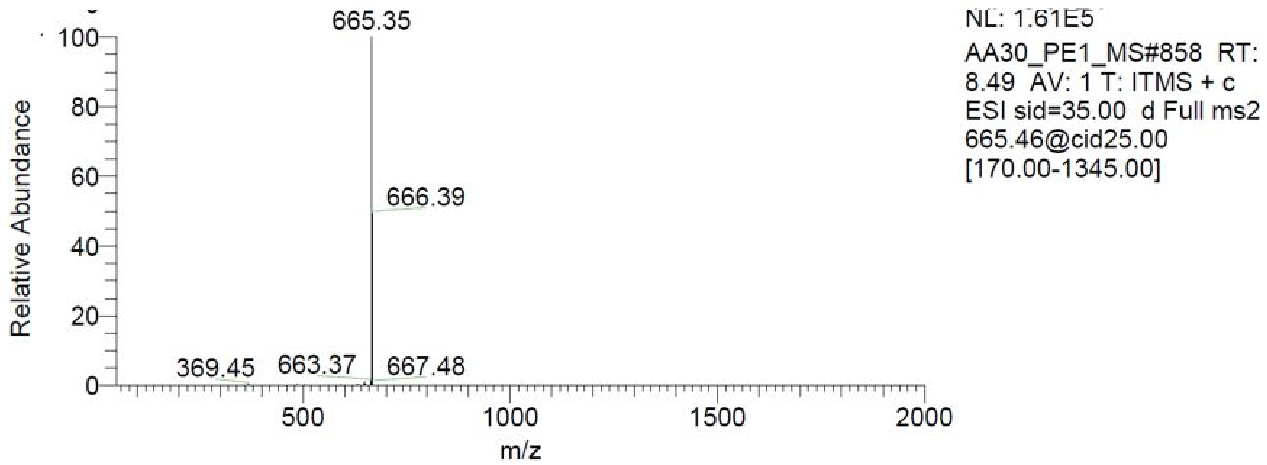

NL: 1.61E5
AA30_PE1_MS#858 RT: 8.49 AV: 1 T: ITMS + c ESI sid=35.00 d Full ms2 665.46@cid25.00 [170.00-1345.00]
Relative Abundance
m/z
665.35
666.39
369.45
663.37
667.48

Compound 9

^{1}H-NMR (300 MHz, $CDCl_3$)

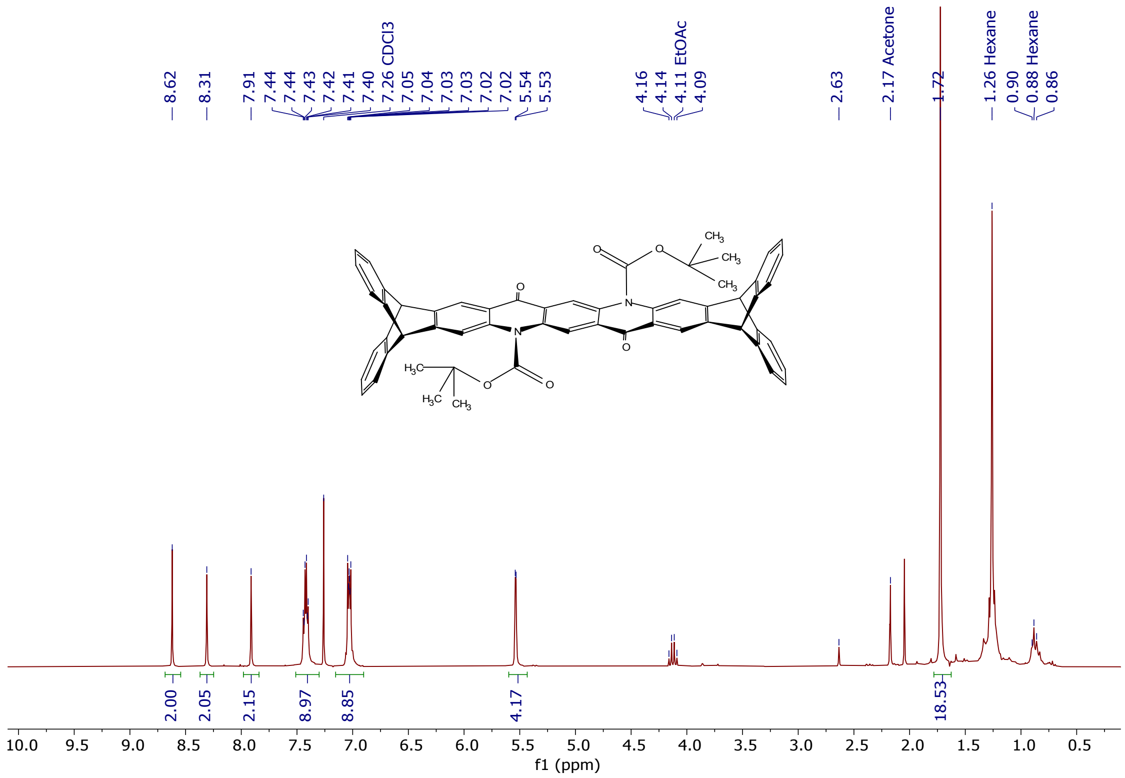

^{13}C-NMR (75 MHz, $CDCl_3$)

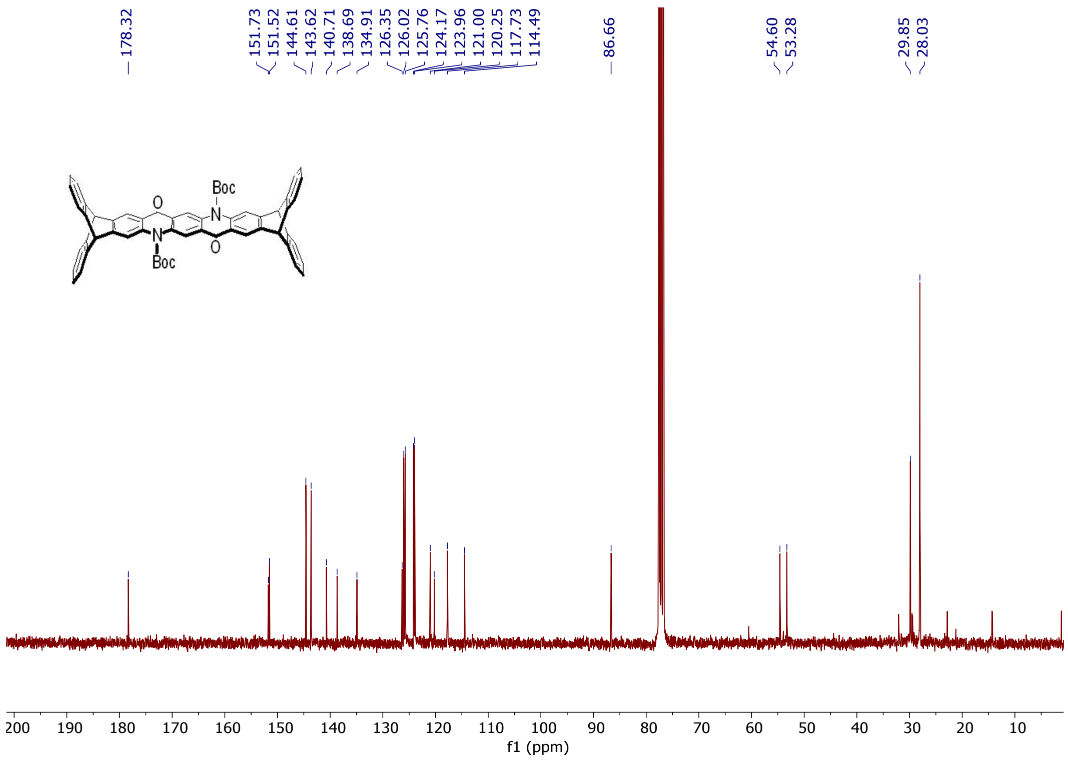

COSY

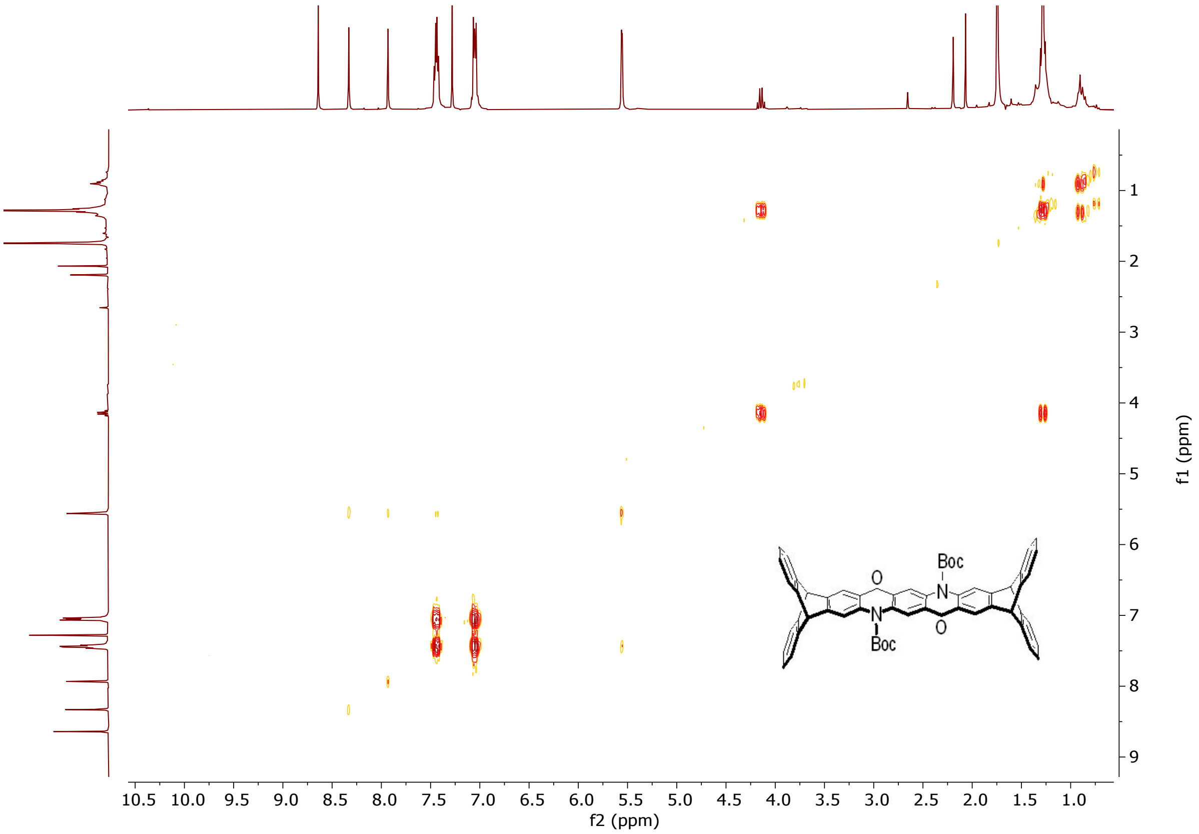

HSQC

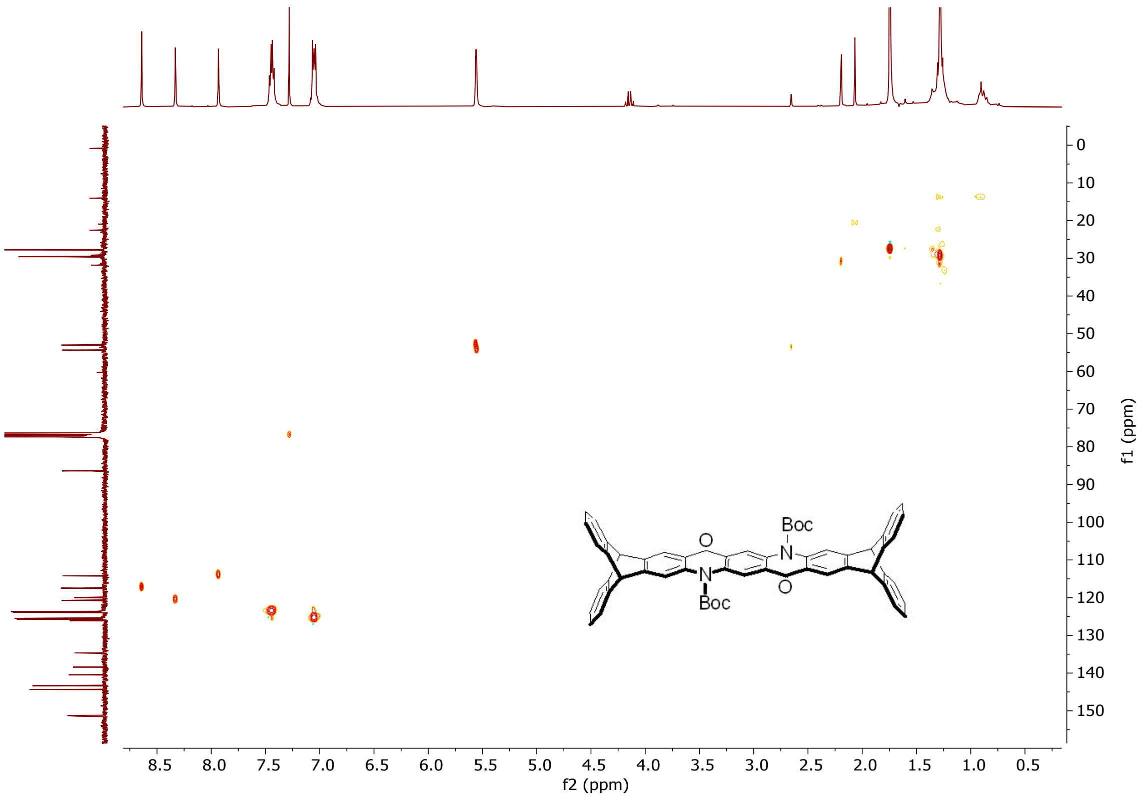

DEP MS

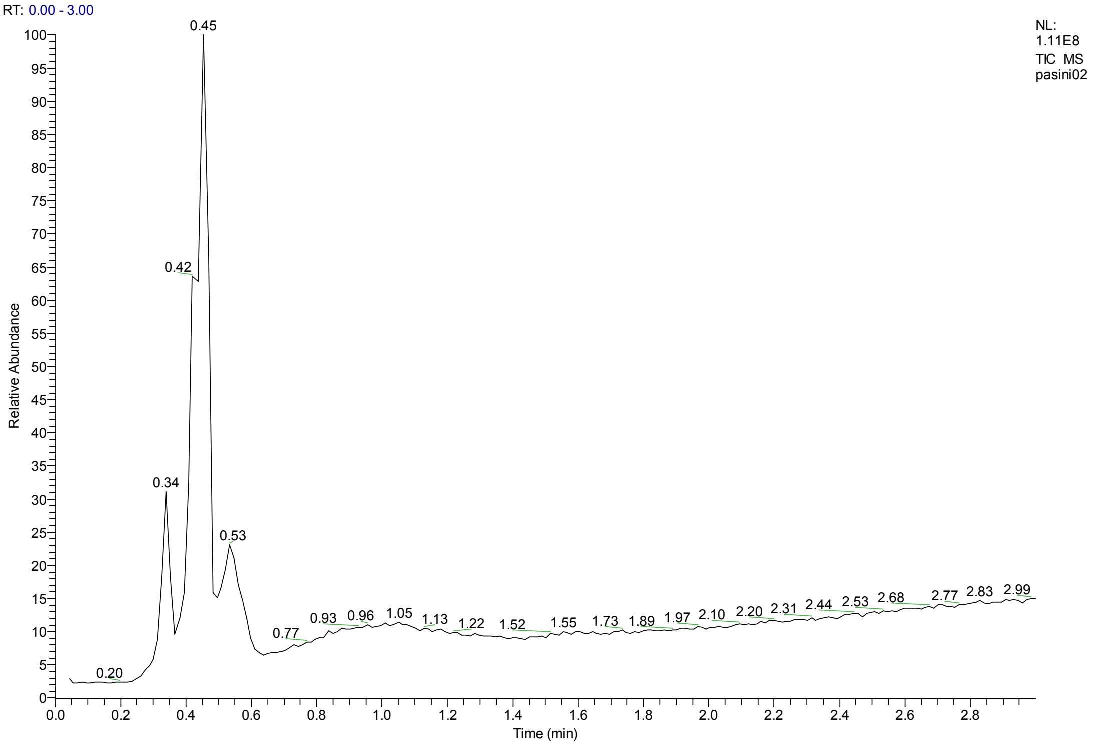
RT: 0.00 - 3.00
NL:
1.11E8
TIC MS
pasini02
Relative Abundance
Time (min)

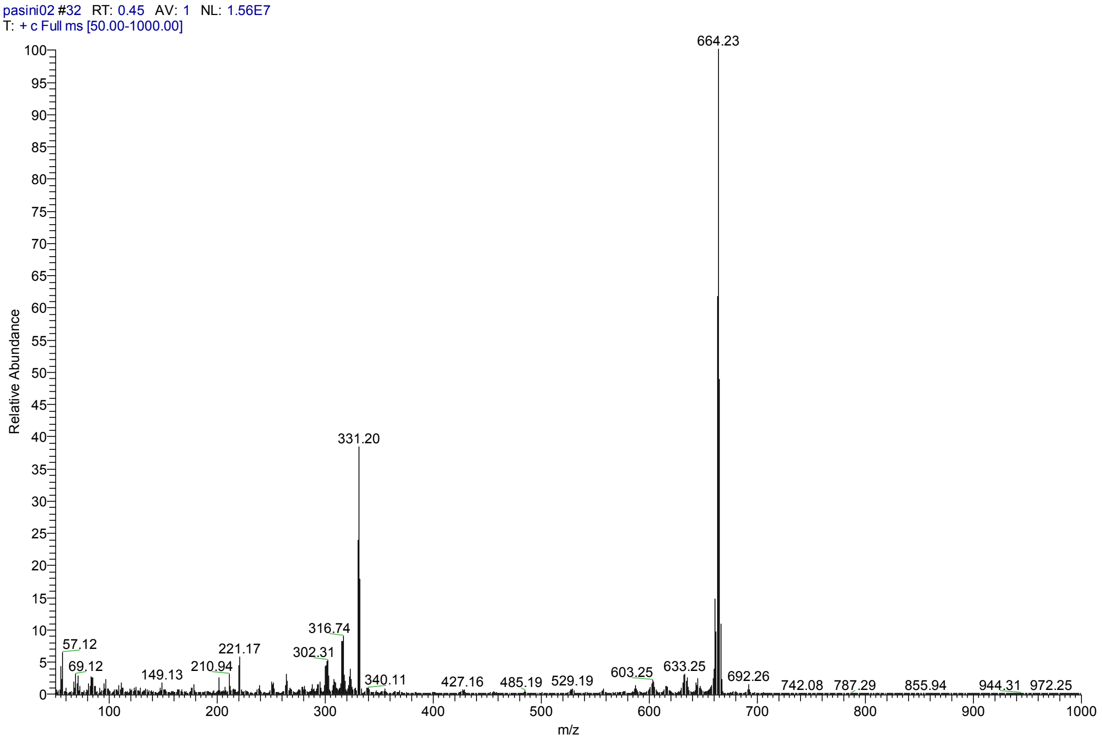
pasini02 #32 RT: 0.45 AV: 1 NL: 1.56E7
T: + c Full ms [50.00-1000.00]
Relative Abundance
m/z

Compound 10

^{1}H-NMR (300 MHz, $CDCl_3$)

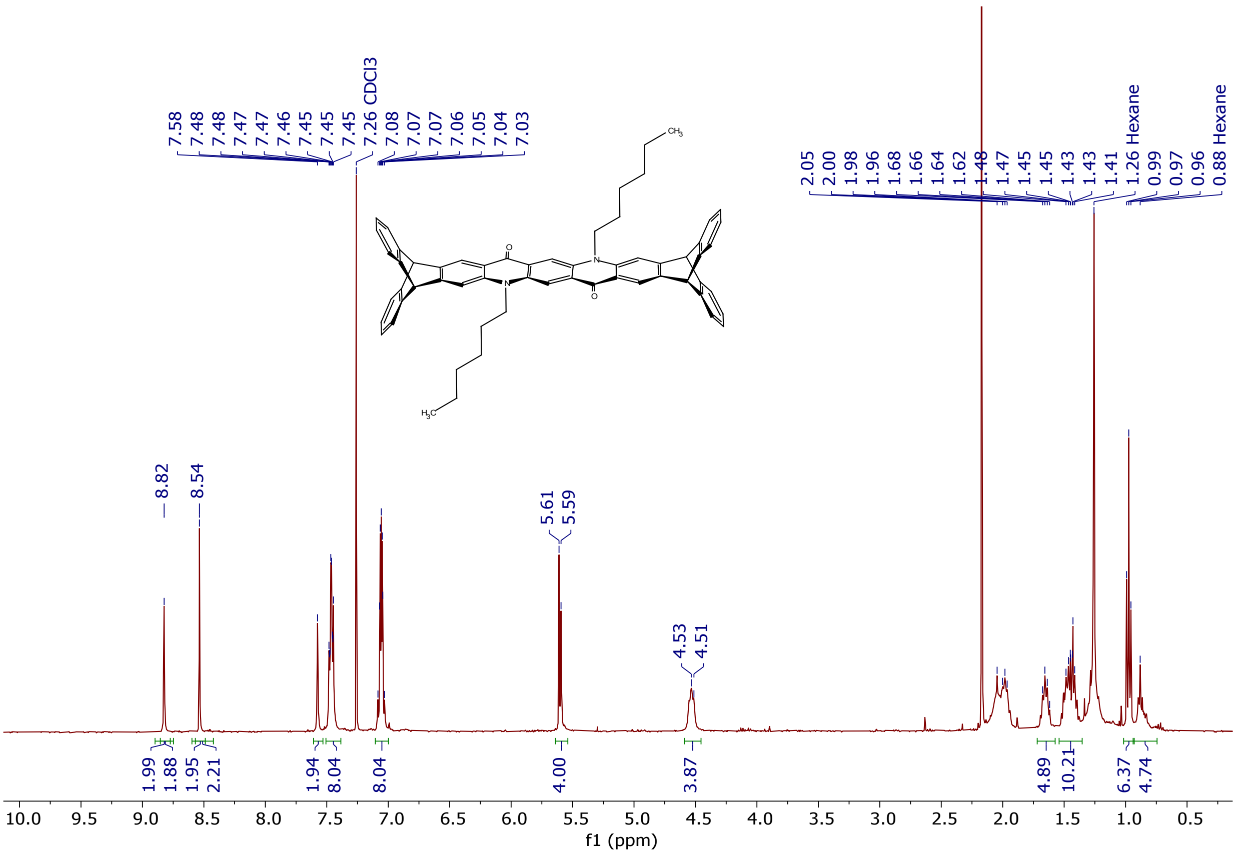

^{13}C-NMR (75 MHz, $CDCl_3$)

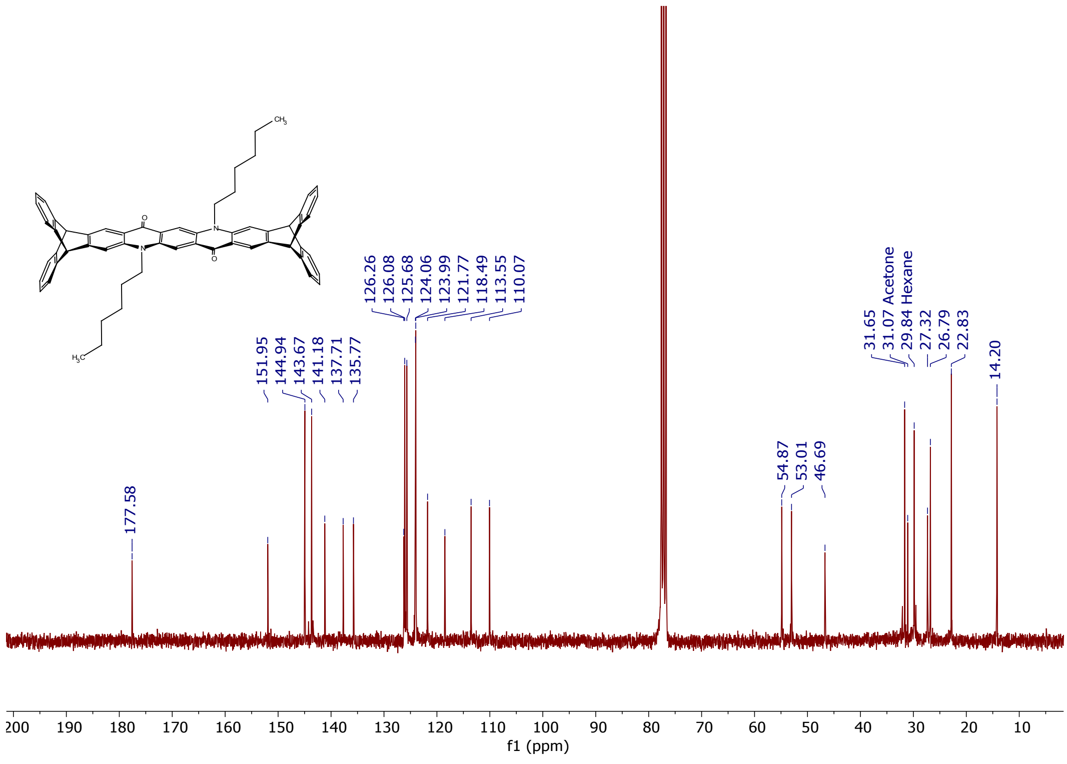

COSY

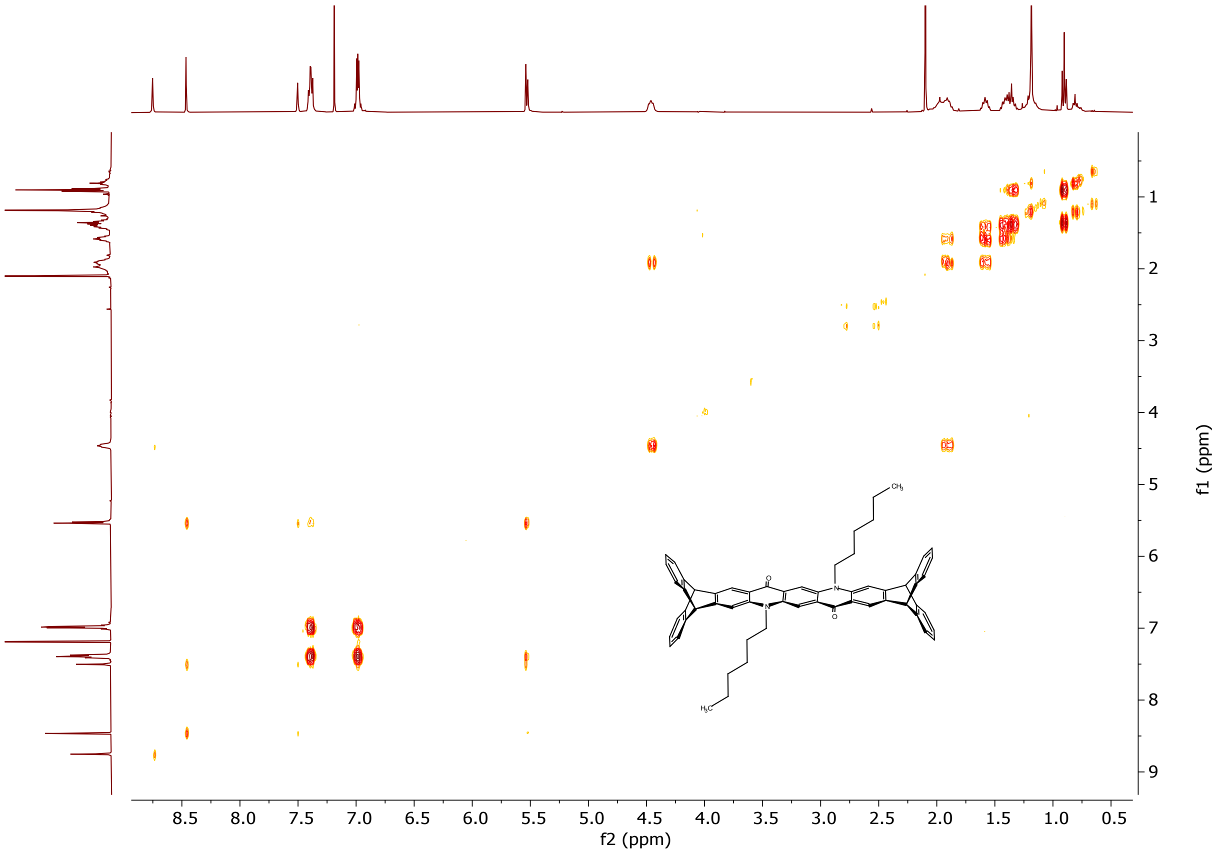

HSQC

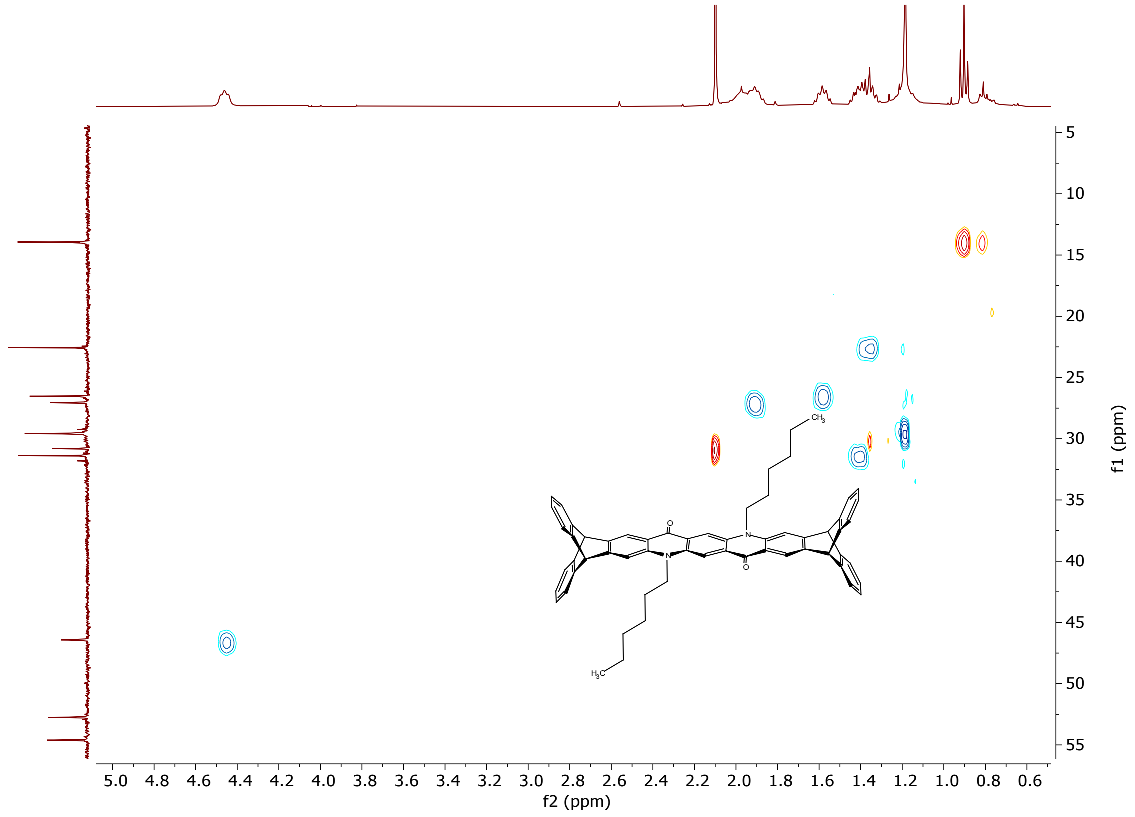

MALDI-TOF spectrum

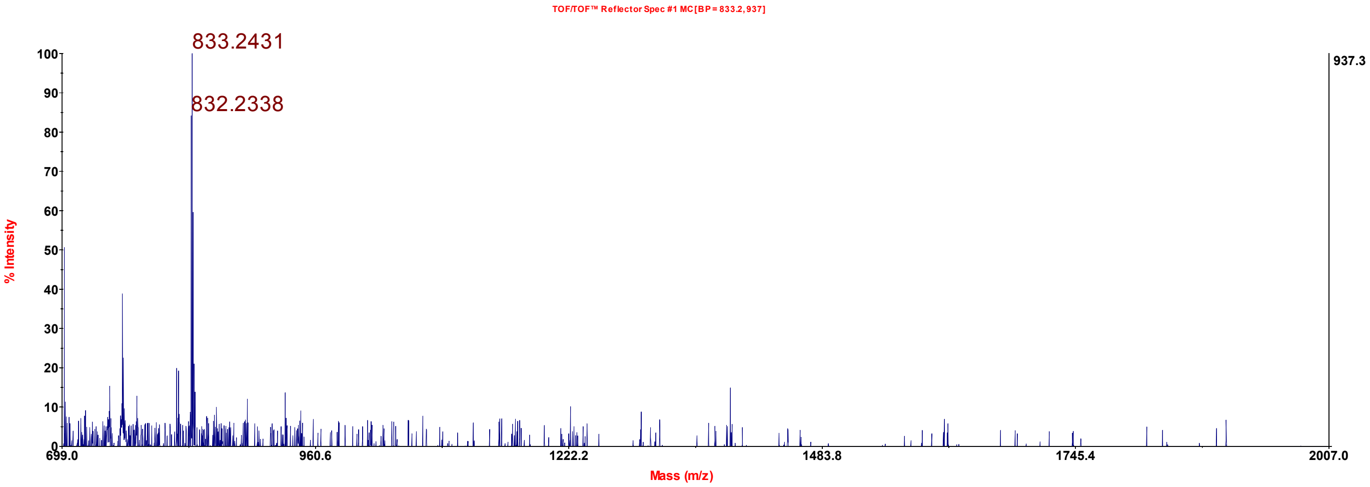

9. Additional references

S1. Granda, J. M., Grabowski, J.; Jurczak, J. Synthesis, Structure, and Complexation Properties of a C3-Symmetrical Triptycene-Based Anion Receptor: Selectivity for Dihydrogen Phosphate. *Org. Lett.* **2015**, *17*, 5882–5885.

S2. Chong, J. H.; MacLachlan, M. J. Robust Non-Interpenetrating Coordination Frameworks from New Shape-Persistent Building Blocks. *Inorg. Chem.* **2006**, *45*, 1442−1444

S3. Huang, W. *et al.* Molecular Design of Deep Blue Thermally Activated Delayed Fluorescence Materials Employing a Homoconjugative Triptycene Scaffold and Dihedral Angle Tuning. *Chem. Mater.* **2018**, *30*, 1462–1466.

S4. Wang, X.; Wang, K.; Xu, J.; Li, J.; Lv, J.; Zhao, M.; Wang, L.. Quinacridone skeleton as a promising efficient leveler for smooth and conformal copper electrodeposition. *Dyes Pigment.* **2020**, *181*, 108594.

S5. Moreau, J.; Giovanella, U.; Bombenger, J.; Porzio, W.; Vohra, V.; Spadacini, L.; Di Silvestro, G.; Barba, L.; Arrighetti, G.; Destri, S.; Pasini, M.; Saba, M.; Quochi, F.; Mura, A.; Bongiovanni, G.; Fiorini, M.; Uslenghi, M.; Botta, C..Highly emissive nanostructured thin films of organic host–guests for energy conversion. *ChemPhysChem* **2009**, *10*, 647.

S6 Mula, S.; Han, T. ; Heiser, T.; Lèveque, P.; Leclerc, N.; Srivastava, A.P.; Ruiz-Carretero, A.; G. Ulrich, A.. Hydrogen bonding as a supramolecular tool for robust OFET devices. *Chem.Eur.J.* **2019**, *25*, 8304 –8312.

S7. Gaussian 16, Revision C.01, Frisch, M. J.; Trucks, G. W.; Schlegel, H. B.; Scuseria, G. E.; Robb, M. A.; Cheeseman, J. R.; Scalmani, G.; Barone, V.; Petersson, G. A.; Nakatsuji, H.; Li, X.; Caricato, M.; Marenich, A. V.; Bloino, J.; Janesko, B. G.; Gomperts, R.; Mennucci, B.; Hratchian, H. P.; Ortiz, J. V.; Izmaylov, A. F.; Sonnenberg, J. L.; Williams-Young, D.; Ding, F.; Lipparini, F.; Egidi, F.; Goings, J.; Peng, B.; Petrone, A.; Henderson, T.; Ranasinghe, D.; Zakrzewski, V. G.; Gao, J.; Rega, N.; Zheng, G.; Liang, W.; Hada, M.; Ehara, M.; Toyota, K.; Fukuda, R.; Hasegawa, J.; Ishida, M.; Nakajima, T.; Honda, Y.; Kitao, O.; Nakai, H.; Vreven, T.; Throssell, K.; Montgomery, J. A., Jr.; Peralta, J. E.; Ogliaro, F.; Bearpark, M. J.; Heyd, J. J.; Brothers, E. N.; Kudin, K. N.; Staroverov, V. N.; Keith, T. A.; Kobayashi, R.; Normand, J.; Raghavachari, K.; Rendell, A. P.; Burant, J. C.; Iyengar, S. S.; Tomasi, J.; Cossi, M.; Millam, J. M.; Klene, M.; Adamo, C.; Cammi, R.; Ochterski, J. W.; Martin, R. L.; Morokuma, K.; Farkas, O.; Foresman, J. B.; Fox, D. J. Gaussian, Inc., Wallingford CT, **2016**.
